# Severe COVID-19 induces prolonged elevation of the acute-phase protein pentraxin 3

**DOI:** 10.3389/fimmu.2025.1672485

**Published:** 2025-10-01

**Authors:** Bernhard Kratzer, Robert B. Stieger, Seyma Durmus, Doris Trapin, Pia Gattinger, Paul Ettel, Al Nasar Ahmed Sehgal, Kristina Borochova, Yulia Dorofeeva, Inna Tulaeva, Katharina Grabmeier-Pfistershammer, Peter A. Tauber, Marika Gerdov, Thomas Perkmann, Ingrid Fae, Sabine Wenda, Michael Kundi, Sebastian Wrighton, Gottfried F. Fischer, Rudolf Valenta, Winfried F. Pickl

**Affiliations:** ^1^ Institute of Immunology, Center for Pathophysiology, Infectiology and Immunology, Medical University of Vienna, Vienna, Austria; ^2^ Institute of Pathophysiology and Allergy Research, Center for Pathophysiology, Infectiology and Immunology, Medical University of Vienna, Vienna, Austria; ^3^ Laboratory for Immunopathology, Department of Clinical Immunology and Allergology, I. M. Sechenov First Moscow State Medical University (Sechenov University), Moscow, Russia; ^4^ Life Improvement by Future Technologies (LIFT) Center, Moscow, Russia; ^5^ Department of Laboratory Medicine, Medical University of Vienna, Vienna, Austria; ^6^ Department of Transfusion Medicine and Cell Therapy, Medical University of Vienna, Vienna, Austria; ^7^ Department for Environmental Health, Center for Public Health, Medical University of Vienna, Vienna, Austria; ^8^ Center for Molecular Allergology, Karl Landsteiner University, Krems, Austria; ^9^ Karl Landsteiner University of Health Sciences, Krems, Austria

**Keywords:** COVID-19, pentraxin-3, soluble pattern recognition receptors, acute-phase proteins, severe COVID-19

## Abstract

**Introduction:**

During the acute-phase of COVID-19, elevated levels of several acute-phase proteins, such as C-reactive protein (CRP), mannose-binding lectin (MBL), pentraxin 3 (PTX-3), serum amyloid A (SAA) and surfactant protein D (SP-D), are associated with severe to fatal clinical outcomes. Typically, these markers return to baseline within days after resolution of the acute infection.

**Methods:**

In this study, we assessed the plasma levels of these proteins in a well-defined cohort of 141 COVID-19 convalescent patients 10 weeks after infection and compared them to 98 non-infected controls. In addition, we performed genetic analyses in a subgroup of patients and related the findings with structural equation modelling to disease severity.

**Results:**

In contrast to other acute-phase proteins, PTX-3 levels were significantly higher in severe COVID-19 convalescent patients than in the control group. Furthermore, a higher proportion of patients with severe COVID-19 exhibited PTX-3 levels above 5000 pg/ml even 10 months post-infection, compared to those with mild disease. To explore potential genetic influences, a genetic analysis was performed on all severely affected patients (n=36) and on an age- and sex-matched subset of mild COVID-19 patients (n=38). Results revealed a significantly higher frequency (p<0.0001) of the homozygous wildtype genotype of the PTX-3 SNP rs971145291 in severe (15 out of 36) versus mild (1 out of 38) COVID-19 patients. Using structural equation modelling, the association of this PTX-3 genotype and disease severity was shown to be mediated by elevated PTX-3 levels, with no contribution from other analyzed (clinical) confounders.

**Discussion:**

In summary, severe COVID-19 patients show high PTX-3 serum levels which may be influenced by genetic predisposition, specifically the absence of the rs971145291 SNP variant. PTX-3 may thus serve both as a biomarker for tissue damage and/or long-term immune activation and eventually post-COVID-19 complications.

## 1 Introduction

The emergence of the β-Coronavirus SARS-CoV-2 in 2019 triggered a worldwide pandemic, leading to millions of severe infections and deaths worldwide (1). Infection with the virus can lead to a wide variety of symptoms, with a considerable portion of patients remaining entirely asymptomatic while others suffer from severe respiratory problems requiring assisted ventilation and admission to intensive care units (ICU). Especially at the onset of the pandemic and prior to the development of effective vaccines, case fatality rates reached up to 5.25% in China (2). Even in non-fatal cases with a generally mild disease course, SARS-CoV-2 infection may leave a long-term negative imprint on the human immune system of those affected (3), which can be detected months after the initial infection (4–8). For instance, in COVID-19 convalescent patients the numbers of recent thymic emigrants (RTE) and granulocytes may be reduced for up to 10 months post-infection in COVID-19 convalescent patients (3, 4, 7).

While the long-term consequences of COVID-19 on adaptive humoral and cellular immunity have been meticulously studied, a possible long-term impact of COVID-19 on innate humoral immune factors, the so-called serum acute-phase reactants or soluble pattern recognition molecules (sPRM), remains understudied as of yet. These molecules represent ancestral predecessors of antibodies, serving as a first line humoral defense during viral and bacterial infections (9). They are increasingly recognized for their ability to bind damage-associated molecular patterns and to induce tissue remodeling (10) – processes that lead to variable increases in their serum concentrations, either by release from preformed stores, or more commonly, by neo-synthesis. Clinically, elevated plasma levels of these proteins can serve as biomarkers for the recognition of acute or chronic disease processes, while their gradual decrease is often interpreted as a sign of recovery and resolution of the inflammatory process. From the broader group of known sPRMs (9), we investigated the long-term impact of COVID-19 on serum levels of the acute-phase reactants CRP, MBL, PTX-3, SAA and SP-D. Elevated levels of any of these five sPRMs during acute SARS-CoV-2 infection/COVID-19 have been variably proposed as predictors of a more severe disease course, and have been associated with increased mortality (11–17).

Among these sPRMs, the long pentraxin, pentraxin-3 (PTX-3, also known as TSG-14) has been identified as a marker during acute COVID-19, that predicts COVID-19-associated pneumonia and, consequently, of a more severe disease course (15, 16, 18). Moreover, it may even directly interact with SARS-CoV-2 proteins (19). PTX-3, is a member of the cyclic multimeric PRMs, commonly referred to as pentraxin superfamily (20–22), which are characterized by a common sequence motif consisting of His-x-Cys-x-Ser/Thr-Trp-x-Ser (23). While PTX-3 is a prototypic member of the long pentraxins, C-reactive protein (CRP) and serum amyloid P component (SAP) belong to the short pentraxins. The latter two are produced primarily in the liver in response to inflammatory stimuli such as interleukin (IL)-6, while PTX-3 is thought to be induced more locally at sites of inflammation or by tissue damage due to the sensing of IL-1β and TNF-α. Furthermore, increased synthesis can be seen as a response to microbial constituents (LPS or fungi) (24, 25) or in small vessel vasculitis (26, 27). Accordingly, possible cellular sources of PTX-3 include epithelial cells, endothelial cells and mononuclear phagocytes (28, 29). At the molecular level, PTX-3 has a quaternary structure characterized by two tetramers linked together by covalent bonds to form an octamer of 340 kDa (30). PTX-3 has been shown to bind to the outer membrane protein A (OmpA) of *K. pneumoniae*, outer membrane vesicles (OMV) of *N. menginitidis*, and nucleocapsid (NC) of SARS-CoV-2 (24). The PTX-3 gene is located on chromosome 3, band q25 and certain genetic variants have been found to correlate with the development of macrophage activation syndrome during COVID-19 (31). The function of PTX-3 is linked to the recognition and binding of infectious agents and damaged cells, eliciting complement activation, improving opsonization as well as leukocyte recruitment to the site of inflammation (23). Serum levels of PTX-3 are rapidly upregulated in response to danger signals, driven by binding sites in its promoter region for inflammatory transcription factors such as PU.1, AP-1, NF-κB, SP1, and NF-IL-6 (23, 32). Compared to CRP, systemic PTX-3 levels have been shown to increase more rapidly in response to infection and/or cell damage, while also resolving more quickly – indicating that PTX-3 is more dynamically regulated than other sPRMs. For instance, after myocardial infarction, PTX-3 levels peak as early as 7.5 hours after hospital admission, while CRP-levels require approximately 24 hours to reach their peak (33, 34). Similar to other sPRMs like CRP, PTX-3 levels have been reported to return to baseline within 3 to 7 days following the acute-phase of infection.

In this study, we investigated the serum levels of the acute-phase reactants/sPRMs CRP, MBL, PTX-3, SAA and SP-D in a well-characterized cohort of mild (n=105) and severe (n=36) COVID-19 cases and compared them with those of a non-infected control cohort (n=98) at 10 weeks post-infection. Differences were found only for PTX-3 and this was further investigated at a later time point, i.e., at 10 months. Notably, even 10 months after infection PTX-3 levels remained high in more patients with severe compared to mild COVID-19. Genetic evaluations revealed that high PTX-3 levels are more frequently associated with genetic predisposition, specifically the absence of the rs971145291 SNP variant in the promoter region, suggesting a pivotal role for PTX-3 during the development and resolution of severe COVID-19.

## 2 Materials and methods

### 2.1 Patients and control subjects

Into this case-control study, 141 patients diagnosed with COVID-19 between May 11, 2020 and October 30, 2020 were enrolled. Venipuncture was performed 84.6 ± 39.8 days after initial diagnosis and a second time 206 ± 15 days after the first visit. Infection was confirmed either by rtPCR test for SARS-CoV-2 (88.7% of patients) and/or by a positive SARS-CoV-2 antibody test (97.9%) (35). The COVID-19 cohort consisted of 105 patients with mild disease, defined as not needing hospitalization, and 36 patients with severe disease, who needed hospitalization during their disease course (**Supplementary Table S1**). Symptoms were assessed using a questionnaire filled out at the time of venipuncture. Of all patients, 123 out of 141 (87.2%) agreed to a second visit and venipuncture around 10 months after their initial diagnosis. In parallel, 98 non-infected control subjects, who were reportedly asymptomatic for the last 10 weeks and who were SARS-CoV-2 negative by certified SARS-CoV-2 antibody testing (Elecsys^®^ Anti-SARS-CoV-2 assay Roche) and had a negative rtPCR test for SARS-CoV-2 at the time of venipuncture were enrolled into the study. This cohort as well as the mild COVID-19 convalescent patients are identical to the one published in our previous reports on the impact of COVID-19 on the immune system (3, 4). Non-infected control subjects and COVID-19 convalescent patients were well matched in terms of demographic data, clinical background and drug intake and consisted of 44 males (44.9%) and 54 females (55.1%) with a median age of 51 years (range 14-77) (**Supplementary Table S1**).

From each patient, EDTA anti-coagulated blood was used for flow cytometric analyses, heparin-anticoagulated blood for cryopreservation of PBMCs and silicon dioxide coagulated serum or EDTA anti-coagulated plasma for serological analyses.

All Participants of the study gave their written informed consent in accordance with the Declaration of Helsinki. The study was approved by the Ethics Committee of the Medical University of Vienna (EK No.: 1302/2020).

### 2.2 ELISA for soluble pattern recognition molecules

For the determination of the plasma levels of CRP, MBL, PTX-3, SAA and SP-D commercially available “DuoSet” ELISA Kits from R&D Systems (Minneapolis, MN, USA) were used. Measurements were carried out according to the manufacturer’s protocols and the plasma levels of sPRM were quantified with a standard curve (also provided by the manufacturer). Dilutions of plasma samples were first tested in pilot experiments. Accordingly, plasma for determination of the CRP levels were diluted 1:1000, for MBL 1:1000, for PTX-3 1:10, for SAA 1:200 and for SP-D 1:30. Abnormally high test values lying outside of the range of the standard curve were again analyzed with a higher plasma dilution. To control for possible variations between individual ELISA plates, three plasma samples with high, medium or low reactivity with the respective analyte were used as internal controls on each plate and the results of different plates were adjusted accordingly. Generally, a similar number of samples from mild and severe COVID-19 convalescent patients and non-infected control individuals, were applied to each ELISA plate.

### 2.3 Complement assays

For the assessment of complement activation activity, the indicated concentrations of PTX-3 (1 µg/ml, 24.39 nM), IgM (0.2 µg/ml, 0.21 nM) or human serum albumin (HSA, 1 µg/ml, 15.04 nM) were coated in 40 µl of PBS per well in half-area ELISA plates (Greiner, Kremsmünster, Austria) at 4°C overnight and their complement activating activity was determined with the “WIESLAB^®^ Complement system Classical pathway assay” (Wieslab, Malmö, SE) with the following modifications: On the following day, plates were washed three times with 150 µl per well of PBS plus 0.05% Tween 20 and blocked with 150 µl per well of PBS plus 0.05% Tween 20 plus 2% HSA at room temperature for 2 hours. Subsequently, a 1:2 dilution series of normal human serum (preserved at -80°C prior to dilution) over 6 steps, i.e., starting from 2.5% until 0.039% in assay diluent (Wieslab, Malmö, SE) was performed and 40 µl of each serum-dilution was added to the pre-coated ELISA plates. Each serum-dilution was applied in duplicates. The serum was incubated at 37°C for 1 hour, washed four times with 150 µl per well of wash buffer (Wieslab, Malmö, SE) and incubated with 40 µl per well of AP-conjugated anti C5b-9 antibody (dilution as provided by the manufacturer, Wieslab, Malmö, SE) while shaking at room temperature for 30 minutes. After additional three washing steps with 150 µl per well using washing buffer (Wieslab, Malmö, SE), 50 µl per well of pNPP containing substrate was added and incubated at room temperature. OD_405_ values were determined on a MultiskanGo ELISA-reader (Thermo Fisher, Waltham, MA) after 30 min, 60 min, 120 min and 240 min.

In indicated experiments, 1:100 diluted serum was used and supplemented either with recombinant PTX-3 or recombinant IL-2 as negative control, both at concentrations of 2000 pg/ml, 200 pg/ml, 40 pg/ml, 20 pg/ml, and 2 pg/ml. All other steps were performed as described.

### 2.4 Sequencing of the PTX-3 locus

DNA spanning the PTX-3 gene and its flanking regions (chr3:157431423-157449058, GRCh38/hg38) was amplified as three overlapping fragments (of approximately 5.7 kb, 6 kb, and 4.7 kb respectively) using long-range PCR (GoTaq Long PCR Master Mix, Promega, WI, USA). The 5’ region was amplified using the primers GAGTCTCACACTGTTGTCTGG (forward, pos157431872) and CGCAGGACGTCGTCCGTGG (reverse pos157437609) at 66°C annealing temperature. The exon region was amplified using AATGCATCTCCTTGCGATTCTG (forward, pos157436933) and TTATGAAACATACTGAGCTCCTC (reverse, pos157442979) at 62°C. The 3’ region was amplified using CCCCTCCCAAGTGCTCTTTA (forward, pos 157440847) and GAGTGCAGTGCCATGATCTG (reverse, pos 157445589) at 60°C. All PCR conditions comprised initial denaturation at 98°C for 2 minutes, followed by 35 cycles of 98°C for 10 seconds, primer-specific annealing temperature for 20 seconds, and 68°C extension for 5 minutes, with final extension at 72°C for 7 minutes.

Amplicons were enzymatically fragmented (NEBNext Fast DNA Fragmentation & Library Prep Set, New England Biolabs GmbH, Ipswich, MA, USA) and size-selected on an agarose gel (E-Gel EX Agarose Gels, Invitrogen, Thermo Fisher Scientific, Waltham, MA). After ligation of sequencing adaptors, purification and selection of sequenceable products, the prepared library was bound to microspheres, and emulsion PCR (ePCR) amplification was carried out using the Ion OneTouch 2 System (Ion Chef System Thermo Fisher Scientific, Waltham, MA). Sequencing was performed on the Ion GeneStudio S5 sequencer (using Ion 520 Chips and Ion S5™ Sequencing Chemistry, Thermo Fisher Scientific, Waltham, MA).

Sequences were aligned to GRCh38/hg38 using Bowtie2, followed by variant calling using Samtools and Bcftools (36). Sequence variations were stored in variant calling format (vcf). Analyses were performed in R using Bioconductor packages for SNP identification and zygosity determination. Association between SNPs and disease severity was assessed using Fisher’s exact test using R (with significance threshold set at p<0.05, using the Bonferoni correction for multiple testing).

### 2.5 Flow cytometric binding assay

Stably transfected HEK 293T cells (2x10^5^) expressing FLAG::ACE2, FLAG::NC::GPI, FLAG::S::GPI, RBD::GPI or wildtype (wt) cells (37, 38) as control were transferred into 4.5 ml polystyrene FACS tubes (BD, Franklin Lakes, NJ), washed two times (500 g, 5 minutes) with 4.5 ml of PBS plus 0.05% BSA plus 0.05% NaN_3_ and incubated with 0.25 µg per tube of 6x-His-tagged PTX-3 (R&D Systems (Minneapolis, MN, USA), 0.25 µg 6x-His-tagged RBD Wuhan-Hu-1 (Genscript, Piscataway, NJ) or anti-ACE2-biotin clone 1.48B (kindly provided by Pablo Engel, Immunology Unit, Department of Biomedical Sciences, Faculty of Medicine and Medical Sciences, University of Barcelona, Barcelona, Spain) at 4°C for 30 minutes. Afterwards, cells were washed as previously described and 20 µl of anti-His-PE (1:50 diluted, Thermo Fisher Scientific, Waltham, MA) Streptavidin-PE (1:100 diluted, Thermo Fisher Scientific, Waltham, MA) or anti-FLAG-PE (1:100 diluted, Thermo Fisher Scientific, Waltham, MA) were added for another incubation step at 4°C for 30 minutes. After a final wash, 10 µl of 0.5 µg/ml DAPI (Sigma Aldrich, St. Louis, MO) was added and at least 1x10^4^ live cells (DAPI negative singlets) were acquired on a FACS Fortessa flow cytometer (BD, Franklin Lakes, NJ) equipped with the DIVA software package (BD, Franklin Lakes, NJ) and analyzed with the FlowJo software (BD, Franklin Lakes, NJ).

### 2.6 Statistical analyses

Statistical differences between mild and severe COVID-19 patients and non-infected controls were assessed by the Kruskal-Wallis tests followed by Dunn’s comparison test for some variables showing outliers or were skewed. Comparison between two groups were in such cases assessed by Mann Whitney U-tests. For variables whose residuals did not deviate from a normal distribution as determined by Shapiro-Wilk test results the differences were analyzed by one-way ANOVA followed by Tukey’s honest multiple comparison tests. Comparison of categorical variables between two groups were performed by Fisher’s exact test. A principal component analysis of acute-phase protein levels resulted in two factors with eigenvalues above 1 that explained 59% of the variance. Duration of disease, days of bed rest, days hospitalized, and ICU days were submitted to a principal factor analysis that revealed a one-factor solution. The weights were obtained from the factor loadings (rounded to two decimals, they were 0.04 for disease duration, 0.45 both for days of bed rest and days hospitalized, and 0.95 for ICU days). The resulting score turned out to be normally distributed. A generalized structural equation model (GSEM) was applied to analyze the relationship between the SNP homozygous wild type configuration (exogenous variable) and disease severity, with PTX-3 as a potentially moderating endogenous variable. Normality of log PTX-3 and disease severity was confirmed by Shapiro-Wilk tests and SNP was modeled as a binomial variable. The a posteriory power of the model to detect the observed effects was above 90%. In order to assess the highly significant correlation between elevated PTX-3 levels and disease severity, a sensitivity analysis was performed to determine whether inclusion of demographic information (age and gender), BMI, acute-phase proteins other than PTX-3, or comorbidities, such as cardiovascular diseases, chronic lung diseases, allergy/asthma, diabetes mellitus, hematopoietic diseases, immunosuppressive conditions, liver diseases, metabolic diseases, neurological disorders, renal diseases or any other medical problem mentioned by the patients but not fitting in the listed groups did not change this relationship. Standardized regression coefficients and their 95% confidence intervals were computed and their changes determined as different variables were introduced into the analysis. For this sensitivity analysis, a modifying effect of the included variables resulting in an increase of R-square of 8% would have been detected with 80% power. For data management MS Excel was used and calculations were performed using GraphPad 10.3 software (GraphPad Software Inc., La Jolla, CA) and Stata 17.0 (StataCorp, College Stations, TX), respectively.

## 3 Results

### 3.1 Significantly higher levels of pentraxin 3, but not of other acute-phase proteins, in the serum of COVID-19 convalescent patients 10 weeks post-infection

**Figure 1:**
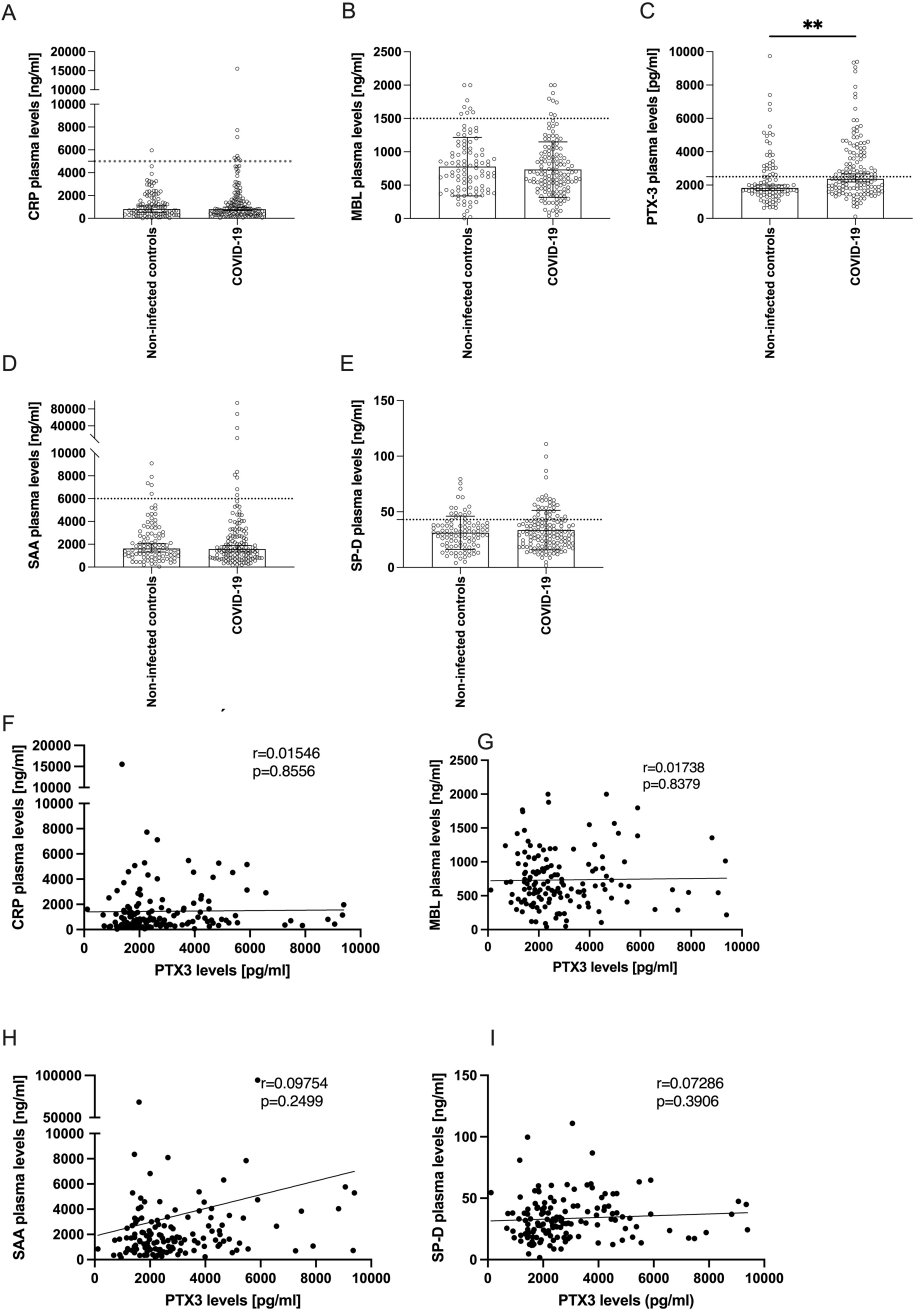
Levels of acute-phase proteins in plasma of COVID-19 patients 10 weeks after infection compared to non-infected controls. **(A-E)** Bars show the median ± 95% confidence interval of plasma levels (y-axes) of **(A)** CRP, **(B)** MBL, **(C)** PTX-3, **(D)** SAA or **(E)** SP-D of non-infected controls (n=98) and COVID-19 convalescent patients 10 weeks after infection (n=141). Horizontal dotted lines indicate the upper limit of the normal range. P-values were determined using the Mann Whitney U-test and are indicated as follows: **p<0.01. Only statistically significant differences are shown. **(F-I)** show correlation analyses between the plasma levels of PTX-3 (x-axes) and the plasma levels (y-axes) of CRP **(F)**, MBL **(G)**, SAA **(H)** and SP-D **(I)**. The Pearson’s r- and p-values are indicated in the respective plot.

To assess the potential role of key soluble pattern recognition molecules CRP, MBL, PTX-3, SAA, and SP-D on COVID-19 convalescence, we measured their serum levels in a cohort of 141 convalescent patients at 10 weeks and 10 months post-infection and compared the results with those of 98 non-infected control individuals (**Figure 1**). Convalescent patients had experienced either a mild (n=105) or severe (n=36) disease course, with severity defined as a score greater than 3 on the WHO clinical progression scale, indicating hospitalization due to COVID-19 (39). The demographic and clinical characteristics, excluding COVID-19-related symptoms, were largely comparable between the COVID-19 convalescent cohort and the non-infected control group. Likewise, the two subgroups of COVID-19 convalescent patients (mild versus severe) were similar, with the exception of age (p=0.0641) and regular medication use (p<0.0001), both of which were higher among patients who had experienced a severe disease course (**Supplementary Table S1**). Although diabetes mellitus (p=0.0474) and immunosuppressive conditions (p=0.0260) were more prevalent in the severe group, the absolute numbers of such cases remained low (**Supplementary Table S1**).

We analyzed serum levels of five sPRMs – CRP, MBL, PTX-3, SAA, and SP-D – previously identified as dynamically regulated acute-phase reactants in various infectious diseases (40–45), including COVID-19 (11–17). These molecules are known to rapidly return to baseline following infection resolution, consistent with their short serum half-lives (CRP: 19h, MBL: 70h, PTX-3: 4h, SAA: 24h, and SP-D: 6h) (40, 46–49). Among the five sPRMs, only PTX-3 levels were significantly higher in convalescent patients 10 weeks post-infection compared to non-infected controls (**Figures 1A–E**). Specifically, COVID-19 convalescents exhibited mean PTX-3 serum levels of 2830 ± 1833 pg/ml (mean ± SD), significantly higher than the 2371 ± 1576 pg/ml observed in controls (p=0.0012) (**Figure 1C**). In contrast, serum levels of CRP, MBL, SAA, and SP-D in convalescent patients were comparable to those in the control group (**Figures 1A–E**) and generally fell within published reference ranges for healthy individuals (20, 50–53). Moderately elevated PTX-3 and SP-D levels above the upper limit of normal were also detected in some control subjects, likely reflecting comorbidity-related processes present in both study cohorts (**Supplementary Table S1**), rather than COVID-19-specific effects. Finally, we investigated whether serum levels of these acute-phase proteins were co-regulated. Univariate linear regression analyses revealed no significant correlations between PTX-3 and CRP, MBL, SAA, or SP-D, in either the convalescent (**Figures 1F–I**) or the control groups (**Supplementary Figure S1**), indicating independent regulation of these molecules. This finding underscores the unexpectedly isolated higher levels of PTX-3 in COVID-19 convalescent patients 10 weeks after infection.

### 3.2 PTX-3 serum levels are highest in severe COVID-19 convalescent patients 10 weeks post-infection

**Figure 2:**
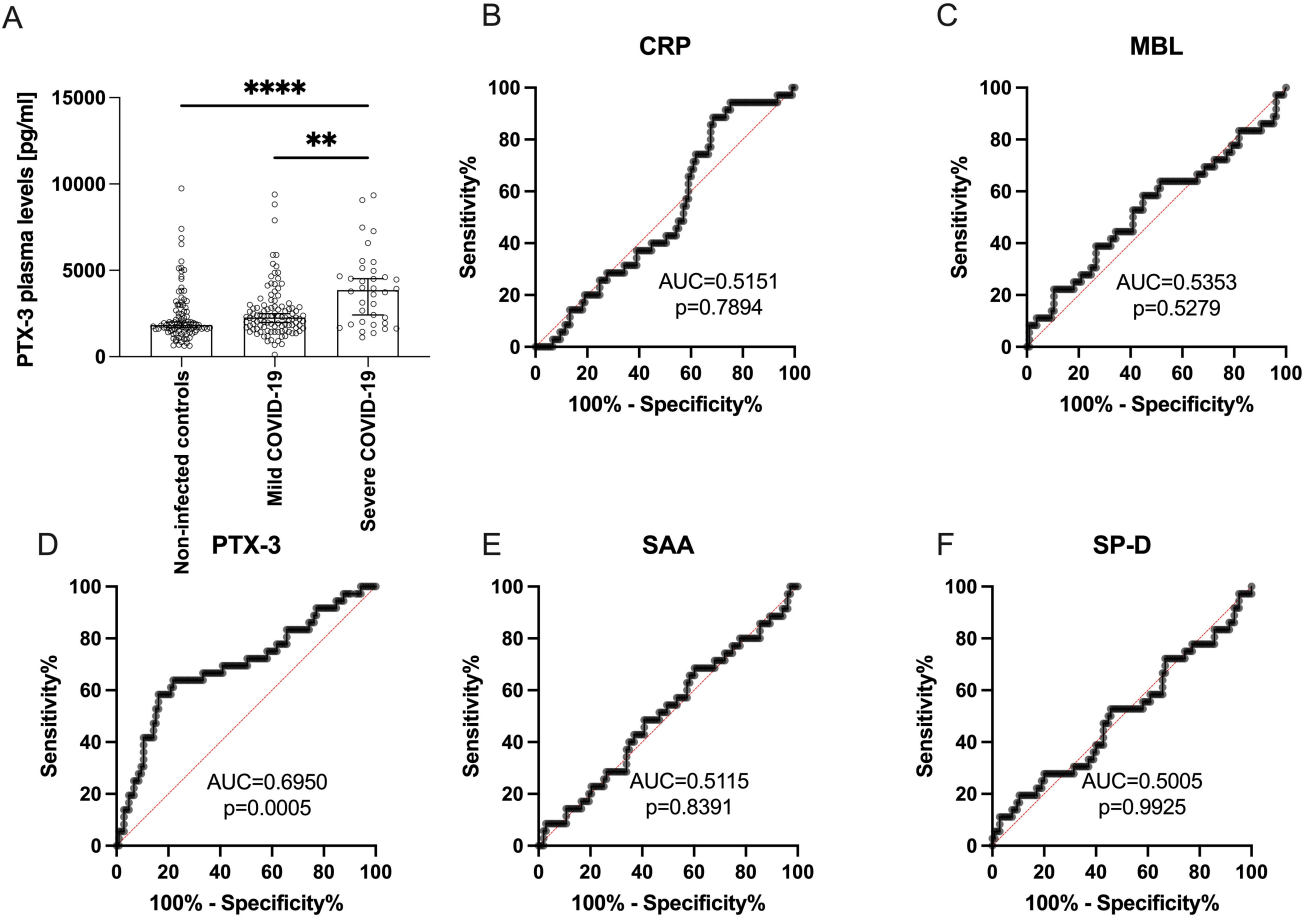
PTX-3 plasma levels are higher in patients 10 weeks after severe SARS-CoV-2 infection as compared to non-infected subjects. **(A)** Shows the PTX-3 plasma levels (y-axes) of 98 non-infected controls, 105 mild and 36 severe COVID-19 subjects (x-axes) 10 weeks after infection. Bars indicate the median ± 95% confidence interval. P-Values were determined using the Kruskal-Wallis test followed by Dunn’s multiple correction test and are indicated as follows: **p<0.01; ****p<0.0001. Only statistically significant differences are shown. **(B-F)** show ROC curves of the plasma levels of **(B)** CRP, **(C)** MBL, **(D)** PTX-3, **(E)** SAA and **(F)** SP-D discriminating between mild and severe COVID-19. Data show the summary of 36 severe and 105 mild COVID-19 convalescent individuals. X-axes show the specificity, y-axes the sensitivity of the individual parameter. AUC, area under the curve. P values indicate the statistical significance.

To investigate whether disease severity influences PTX-3 serum levels, we stratified COVID-19 convalescent patients according to the WHO Clinical Progression Scale, which classifies mild cases as non-hospitalized (score ≤3). Patients with severe disease exhibited significantly higher mean PTX-3 serum levels (3993 ± 2133 pg/ml) than those with mild disease (2618 ± 1700 pg/ml, p=0.0053) at 10 weeks post-infection. Both groups, however, displayed higher PTX-3 levels compared to the non-infected control cohort (2371 ± 1576 pg/ml) (**Figure 2A**). To validate these findings, receiver operating characteristic (ROC) curve analyses were performed for all five sPRMs, using the disease severity as the classification parameter (**Figures 2B–F**). PTX-3 demonstrated a strong ability to differentiate between mild and severe COVID-19 cases 10 weeks after infection, with high significance (AUC 0.6950, p=0.0005) (**Figure 2D**). Collectively, these results indicate that persistently higher PTX-3 serum levels may serve as a retrospective biomarker for severe COVID-19. In addition to the univariate regression analyses, we performed dimensionality reduction using principal component analysis to explore whether shared factors among the soluble pattern recognition molecules – such as activating cytokines, production sites, co-regulated transcription factors, or similarities in promoters/enhancers – could account for the variance observed in the study cohort (**Supplementary Table S2**). These analyses revealed that SAA and CRP clustered on one factor, whereas PTX-3 and SP-D clustered on a separate factor.

The finding of higher PTX-3 serum levels 10 weeks after SARS-CoV-2 infection led us to further examine PTX-3 levels at a later time point, specifically 10 months post-infection.

### 3.3 Certain COVID-19 convalescent patients maintain high PTX-3 serum levels 10 months post-infection

**Figure 3:**
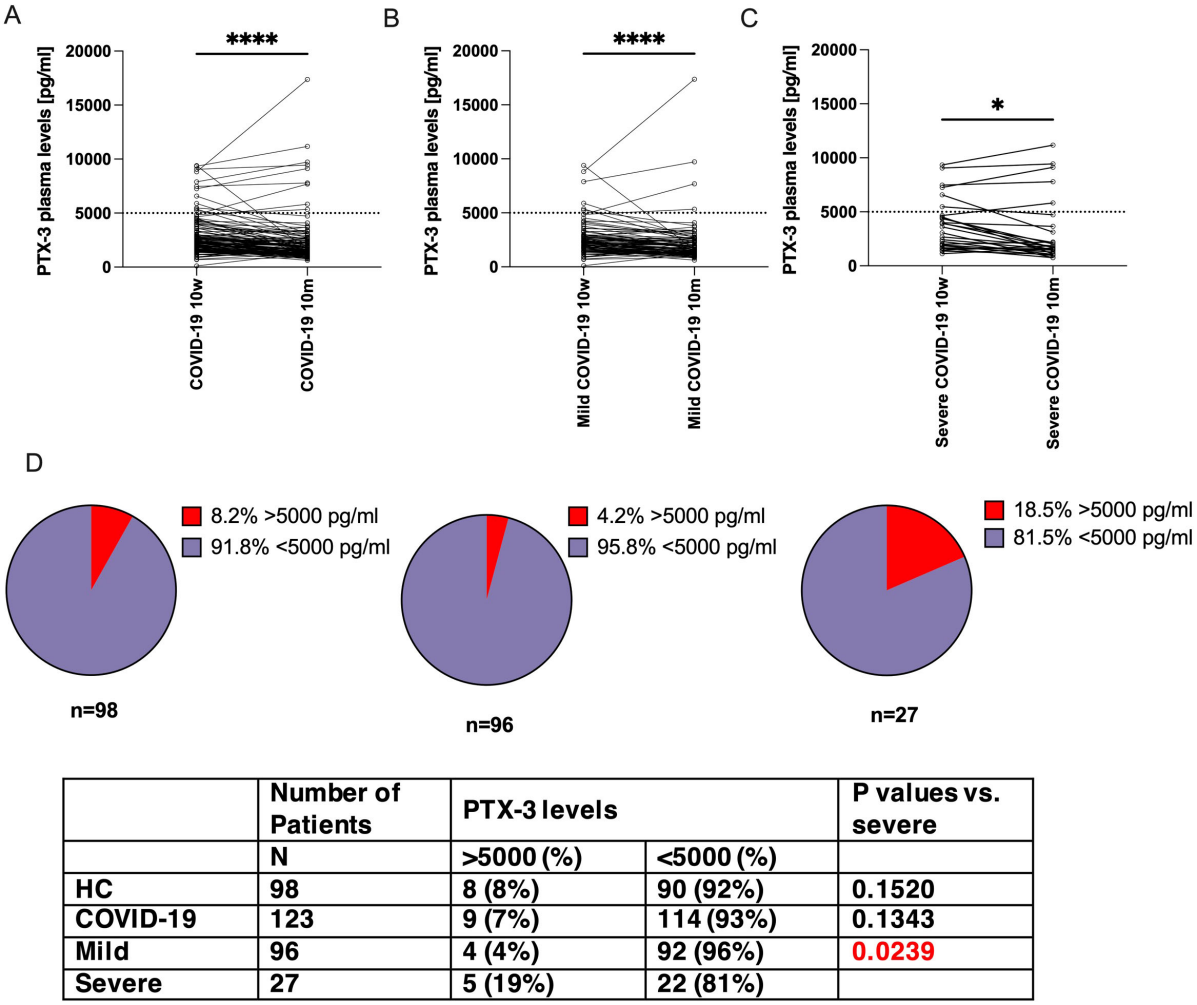
PTX-3 levels decreased more markedly between 10 weeks and 10 months in patients with mild COVID-19 as compared to those with severe COVID 19. Shown are the PTX-3 plasma levels (y-axes) of **(A)** all COVID-19 convalescent patients, **(B)** mild cases or **(C)** severe cases at 10 weeks and 10 months after infection. The same individuals are interconnected by lines. Dotted horizontal line indicates a PTX-3 plasma level of 5000 pg/ml. P-Values were determined using the Wilcoxon rank sum test and are indicated as follows: *, p<0.05; ****, p<0.0001. **(D)** Shows pie charts which indicate the fraction of patients with >5000 pg/ml (red pieces) of PTX-3 in non-infected control individuals, mild and severe COVID-19 case (left to right), at 10m after infection. N indicates the number of patients in the respective group. P-values were calculated with Fisher’s exact test and compared to severe cases.

At 10 months post-infection, PTX-3 serum levels in the overall COVID-19 convalescent cohort had declined to values comparable to those of the control group (**Figure 3**). Among the 123 of 141 individuals who completed both follow-up visits, mean PTX-3 levels decreased from 2830 ± 1833 pg/ml at 10 weeks to 2354 ± 2286 pg/ml at 10 months (**Figure 3A**). Normalization of PTX-3 levels was observed in both patient subgroups. Individuals with a mild disease course showed a reduction from 2618 ± 1700 pg/ml to 2234 ± 2161 pg/ml (mean decrease: 389 ± 1484 pg/ml; 1.34 ± 0.69-fold) (**Figure 3B**, n=96), while those with severe disease showed a decline from 3993 ± 2133 pg/ml to 3014 ± 2858 pg/ml (mean decrease: 815 ± 1442 pg/ml; 1.66 ± 0.90-fold) (**Figure 3C**, n=27), with the difference between groups not reaching statistical significance (p=0.1916).

However, a distinct subset of patients with severe COVID-19 continued to exhibit high PTX-3 serum levels (>5000 pg/ml) at 10 months, with a significantly higher prevalence than in the mild disease group (18.5%, 5 out of 27, versus 4.2%, 4 out of 96, p=0.0239, **Figure 3D**). This proportion was comparable to that observed at 10 weeks (22.2%, 6 out of 27, versus 6.3%, 6 out of 96, p=0.0234). Furthermore, all five individuals with persistently high PTX-3 levels at 10 months had already shown high levels at 10 weeks (range 4500-11181 pg/ml). Notably, in each of these patients, PTX-3 concentrations increased further between the two time points: 4662 → 5817 pg/ml, 7264 → 9112 pg/ml, 7477 → 7789 pg/ml, 9064 → 9434 pg/ml, 9345 → 11,181 pg/ml.

### 3.4 Severe COVID-19 patients more frequently carry the wild type A/A genotype at SNP rs971145291 in the PTX-3 gene

**Figure 4:**
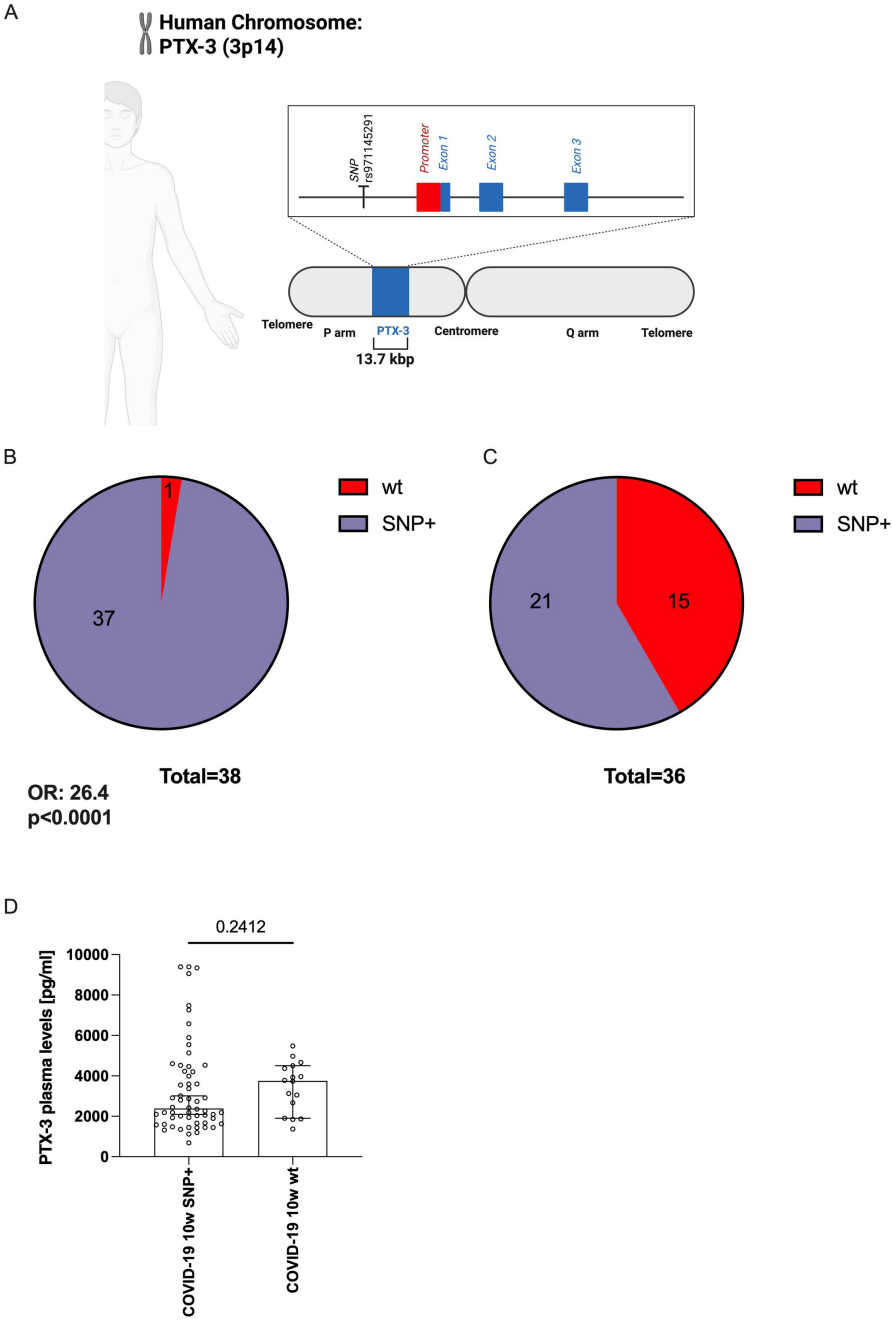
The genetic variant (SNP) rs971145291 is less frequently detected in COVID-19 patients with elevated PTX-3 levels. **(A)** Scheme indicating the position of the SNP rs971145291 relative to the proposed promoter region encoding sequences of the PTX-3 gene. The line shows the 13.7 kbp fragment of the human genome, which contains the PTX-locus and which was sequenced in the patients. The vertical line indicates the position of SNP rs971145291 at bp 2,905 in the sequenced amplicon. The red box indicates the location of the proposed promoter region, covering bp 4,111 to 5,428, the blue boxes indicate the three exons covering bp 5428-5641, 6091–9492 and 10944-12211, respectively. B and C shows pie charts of the PTX-3 locus composition of n=38 mild COVID-19 subjects **(B)** who are mostly (37 out of 38) composed of a G-heterozygous locus and of n=36 severe cases **(C)** in whom only 21 of 36 show the G-heterozygous locus at position rs971145291. **(D)** Shows the median ± 95% confidence interval of the plasma PTX-3 levels (y-axes) of the sequenced subjects (n=74) who show the G-heterozygous locus at position rs971145291 or the wildtype genotype (A/A).

The persistently high serum concentrations of PTX-3 in COVID-19 convalescent patients were unexpected. If these high levels were solely a consequence of SARS-CoV-2 infection, PTX-3 levels would be expected to normalize over time – similar to the other four acute-phase proteins studied. This observation prompted us to investigate whether genetic variations within the PTX-3 locus itself – possibly influencing its transcription – could contribute to sustained high PTX-3 levels or directly impact disease severity. To this end, we sequenced a 13.7 kbp region of the PTX-3 gene on chromosome 3p14 (positions 157431872 to 157445589), which includes all three exons, extensive portions of the 5’- and 3’-UTRs, and both introns (**Figure 4A**). The analysis was performed on samples from 36 convalescent patients with severe COVID-19 and 38 age- and sex-matched patients with mild disease (**Supplementary Figure S2**, **Supplementary Table S3**). Sequencing coverage was >200-fold for most regions, except for a 500 bp fragment (positions 157437500-157438000) where coverage exceeded 20-fold. This analysis confirmed the presence of 45 of the 46 previously identified SNPs in the PTX-3 gene (https://www.ncbi.nlm.nih.gov/snp/) (**Supplementary Table S4**). All identified SNPs, except rs971145291, were similarly distributed between patients with mild or a severe disease (**Supplementary Table S4**). Notably, the wild type (wt) A/A genotype at SNP rs971145291 was strongly associated with severe disease: it was present in 15 of 36 patients with a severe disease course but in only 1 of 38 patients with mild disease (odds ratio [OR] 26.4; 95% confidence interval [CI] 4.2-285.5; p<0.0001; **Figures 4B, C**). Given this strong association with disease severity, we next assessed whether, the A/A genotype correlated with actual PTX-3 serum levels at 10 weeks. Surprisingly, only a moderate association was observed between the wt A/A genotype and PTX-3 levels at 10 weeks (**Figure 4D**), despite the strong relationships observed between PTX-3 serum levels and disease severity, as well as between PTX-3 genotype and disease severity.

### 3.5 The G/A genotype at rs971145291 is associated with more mild COVID-19, potentially through lower PTX-3 serum levels

To determine whether the PTX-3 genotype influences disease severity through its effect on PTX-3 serum levels, we applied a generalized structural equation model (GSEM), treating PTX-3 serum concentrations as an endogenous variable.

**Figure 5:**
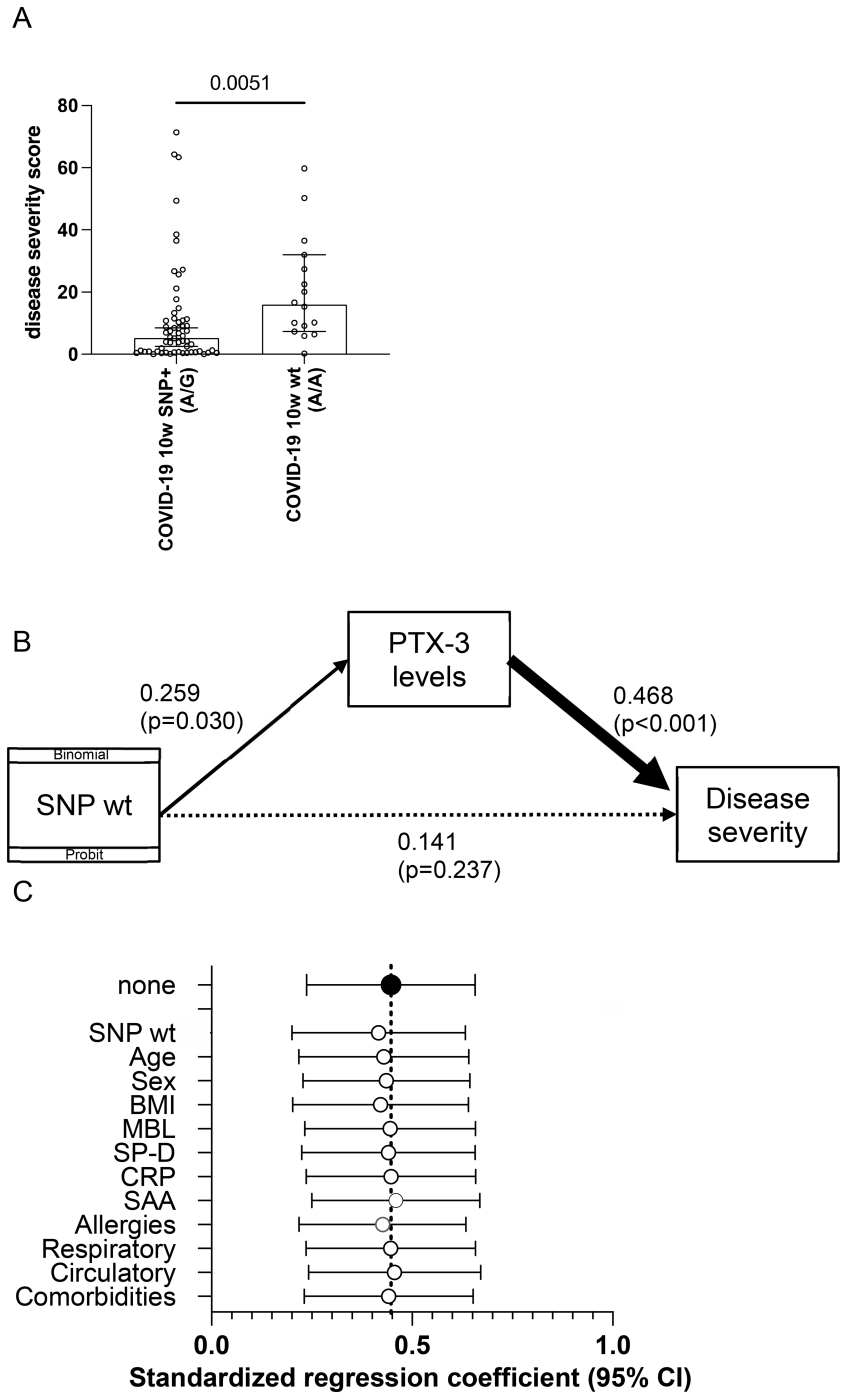
Structural equation modeling reveals an association of the homozygous wt rs971145291 PTX-3 genotype with PTX-3 plasma levels and disease severity. **(A)** Shown is the weighted sum of the disease severity score (y-axis) in patients heterozygous or homozygous (wt) for the SNP locus rs971145291. P-value was calculated with a Mann Whitney U-test and is indicated. **(B)** Shown are the results of structural equation modeling (GSEM), in which the presence of the SNP was used as binomial/probit distribution. **(C)** Shown are the results of sensitivity analyses, which indicate the standardized path regression coefficients +95% confidence intervals (x-axis) possibly contributing to the disease severity score.

Disease severity was quantified using principal factor analysis, which generated a one-factor solution representing a weighted composite score. This score incorporated sick days (weight: 0.04), bed and hospital days (each weight: 0.45), and days in intensive care (weight: 0.95). The resulting severity score followed a normal distribution and was significantly higher in patients who had experienced a severe disease course (**Figure 5A**). GSEM analysis revealed a significant positive effect of the homozygous wt (A/A) genotype on PTX-3 serum levels (standardized path coefficient of β = 0.259, p = 0.0300) and a highly significant effect of PTX-3 serum levels on disease severity (β = 0.468, (p < 0.0010; **Figure 5B**). However, the direct effect of the A/A genotype on disease severity was attenuated and no longer statistically significant (β = 0.141, p = 0.237; **Figure 5B**). These findings indicate that PTX-3 serum levels partially mediate the relationship between the rs971145291 wt genotype and severe COVID-19 outcomes.

To further examine the robustness of the relationship between PTX-3 serum levels and disease severity, we conducted a sensitivity analysis adjusting for demographic variables (age, gender), BMI, serum levels of other acute-phase proteins, and comorbidities (circulatory, respiratory or allergic conditions). Standardized path regression coefficients and their 95% confidence intervals remained consistent across all models (**Figure 5C**), demonstrating that the association between PTX-3 and disease severity was not confounded by these factors. Finally, the small number of subjects with immunosuppression (3 versus 5) or diabetes (4 versus 5) in the control and disease groups, respectively had no measurable impact on the outcomes.

Collectively, these findings support the conclusion that PTX-3 levels mediate disease severity and are, at least in part determined by the SNP configuration at rs971145291, with the presence of the G allele being associated with lower PTX-3 levels and a milder disease course.

### 3.6 Solid-phase bound PTX-3 activates the classical complement pathway

**Figure 6:**
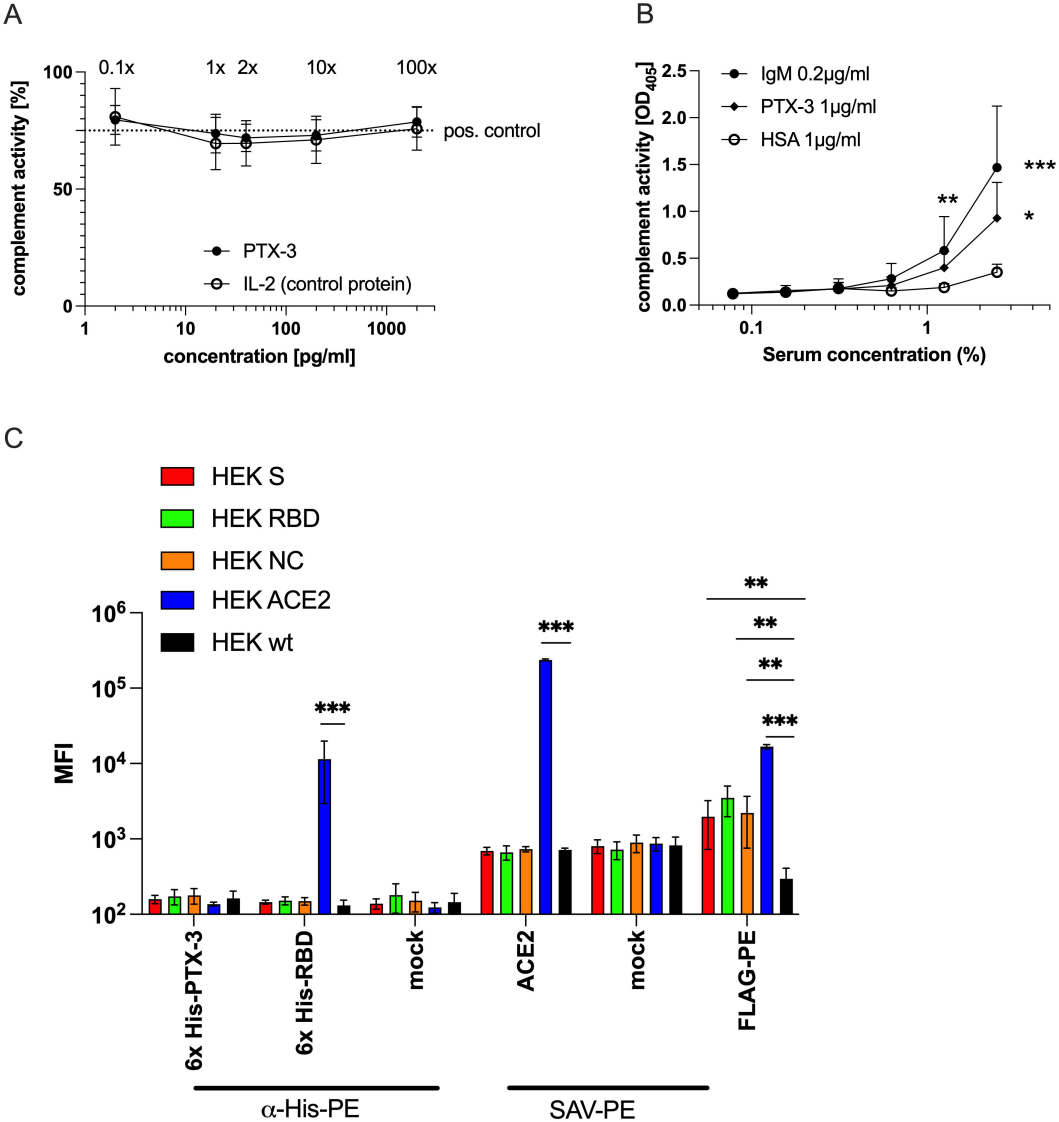
Solid-phase bound, but not soluble PTX-3 can activate complement but shows no direct binding to cellular RBD or NC. **(A)** Shown is the summary data of the relative activity of the classical complement pathway (y-axis) in 1:100 diluted freshly drawn serum of three different healthy donors in the presence of the indicated concentrations (x-axis) of soluble rPTX-3 or rIL-2, used as negative control protein, as determined by a modified classical complement detection kit (Wieslab, Malmö, Sweden). The x-fold of the mean physiological PTX-3 levels are indicated above the respective data points. The original assay uses plate-bound IgM and reads out the C1q-triggered activity of the classical complement pathway leading to the formation of the membrane attack complex, which is detected by a neo-epitope specific AP-labeled anti-human C5b-9 antibody followed by addition of a PNPP-based substrate and determination of the absorbance at OD_405_ nm. The dotted line indicates the level of activity of the positive control supplied by the manufacturer. **(B)** Shows the summary data of classical complement pathway activity (y-axis) in the sera of 5 different healthy individuals at the indicated serum concentrations (x-axes). The wells of ELISA plates were coated with IgM (0.2 µg/ml), PTX-3 (1 µg/ml) or HSA (1 µg/ml) as control, followed by blocking with PBS-T+2% HSA and incubation with the indicated concentrations of human sera. After washing, detection of the formation of membrane attack complex was determined as described above. P-values were calculated with one-way ANOVA followed by Tuckey’s correction for multiple comparisons and the statistical significance against HSA is indicated as follows: *p<0.05; **p<0.001; ***p<0.0001. **(C)** Shows geometric mean fluorescence intensity values (MFI, y-axis) of the indicated HEK-293T cell lines (HEK-293T wt, black; HEK-293T FLAG::ACE2, blue; HEK-293T-FLAG::NC::GPI, orange; HEK-293T FLAG::S::GPI, red and HEK-293T FLAG::RBD::GPI, green) stained with the indicated reagents and/or secondary antibodies (x-axis). Data show the summary of 3 independent experiments. P-values were calculated with Mann Whitney U-test and the statistical significance against wt cells is indicated as follows: **p<0.001; ***p<0.0001.

To investigate whether PTX-3 serum levels could promote inflammation through antibody-dependent or antibody-independent mechanisms (54–58), we conducted a complement activation assay assessing the influence of soluble PTX-3 on C5b-C9 deposition. Plate-bound IgM served as a trigger for complement activation, while fresh serum from healthy donors was used as a source of complement components. The addition of recombinant PTX-3 (rPTX-3), even at concentrations 100-fold higher than physiological levels, did not enhance classical complement deposition compared to controls. Likewise, recombinant IL-2 (rIL-2), used as a negative control, had no effect, even when added at 50,000-fold higher concentrations to 1:100 diluted serum (**Figure 6A**). These findings indicated that soluble PTX-3 does not activate the classical complement pathway.

We next examined whether PTX-3 can activate complement when immobilized, as previously described (58). Microtiter plates were coated with PTX-3 (1 µg/ml), IgM (0.2 µg/ml, positive control), or HSA (1µg/ml, negative control) and incubated with 1.25% or 2.5% fresh serum. Under these conditions, solid-phase PTX-3 significantly activated the classical complement pathway, while HSA did not. Complement activation induced by PTX-3 approached levels observed with coated IgM (**Figure 6B**). It is noteworthy that, on a molar basis, PTX-3 at 1 µg/ml (24.2 nM) was >100-fold more concentrated than IgM at 0.2 µg/ml, which is among the strongest natural activators of the classical pathway. Differences in protein binding efficiency to the ELISA plates – potentially affected by charge, isoelectric point, size, structure, and stability – were not accounted for. Nevertheless, these experiments demonstrate the capacity of immobilized PTX-3 to activate complement deposition.

Previous studies have proposed that PTX-3 may directly bind SARS-CoV-2 proteins (RBD, NC) on viral particles or infected cells (19), potentially enabling its immobilization and subsequent complement activation. However, in our assays, PTX-3 showed no binding to SARS-COV-2 S, RBD, or NC proteins, nor to ACE2-expressing cell lines (37, 38). In contrast, both recombinant RBD and an ACE2-specific monoclonal antibody bound specifically to ACE2 but not to other transfectants (**Figure 6C**), confirming the specificity and reliability of the assay components.

## 4 Discussion

In this study, we report the impact of severe COVID-19 on the innate humoral immune system, with a focus on its effects on the plasma levels of the acute-phase proteins CRP, MBL, PTX-3, SAA, and SP-D. During the pandemic, elevated levels of these molecules were consistently found to be associated with more severe and often more fatal disease outcomes (11–17). In our study, the mean serum concentrations of these acute-phase proteins in the non-infected control population were consistent with values previously reported in the literature (20, 50–53) (**Figures 1A–E**). Following resolution of an infection, the levels of all assessed molecules are expected to return to baseline within 2 weeks. We compared plasma levels among 98 non-infected controls, 105 individuals with mild COVID-19, and 36 individuals who experienced a severe disease course, defined by a WHO clinical progression scale score >3, primarily indicating hospitalization (39). Notably, when assessing PTX-3 levels 10 weeks after acute infection, they were particularly high in individuals with a severe disease course but there was no difference between non-infected and mild COVID-19 patients. In contrast, CRP, MBL, SAA and SP-D levels were generally within the normal range (see **Figure 2A**). Accordingly, PTX-3 levels can be considered as a biomarker for severe COVID-19 (**Figure 2**), and may retrospectively, provide insight into the severity of past infections. As possible confounders for the elevated PTX-3 levels, the only difference between the mild and severe COVID-19 convalescent patients were age (p=0.0641) and regular medication use (p<0.0001), which were higher in patients who suffered from a severe disease course (**Supplementary Table S1**). With regards to comorbidities, significantly more individuals in the severe group suffered from diabetes mellitus (p=0.0474) or immunosuppressive conditions (p=0.0260), but the overall number of such cases was low in both groups (**Supplementary Table S1**). A sensitivity analysis confirmed that the confounders (age, gender, BMI, serum levels of other acute-phase proteins or comorbidities, such as circulatory, respiratory or allergies) did not contribute to the mediation of PTX-3 and disease severity (**Figure 5C**). With regards to regular medication intake, no statistical difference in the PTX-3 levels of patients after severe COVID-19 with and without regular medication intake 10w after infection was found, although future studies with higher patient numbers need to confirm these findings. Whether PTX-3 also serves as a marker for long COVID-19 remains to be determined; however, this question lies beyond the scope of the present study. Since we and others demonstrated that PTX-3 is a critical regulator of complement activity and dysregulated complement activity may be one of the pathomechanisms of long-COVID-19, it is tempting to speculate that elevated serum levels of PTX-3 may critically contribute to the development of long-COVID-19. To the best of our knowledge, so far, only one study reported elevated PTX-3 serum levels as being associated with long COVID-19 (59), while two other reports did not find such changes of PTX-3 serum levels in long-COVID-19 patients at all (17, 60) or rather noted elevated PTX-3 serum levels to be associated with long-term immune recovery from COVID-19 (17). These seemingly conflicting results may be the results of different definitions used to characterize the long-COVID-19 cohorts in the different studies and of the different timepoints at which PTX-3 levels after acute infection were analyzed and highlight the urgent need for further studies clearly stratifying these important study parameters in that respect. In contrast to the fostering of excessive immune responses, PTX-3 might also have the capacity to limit inflammation, for example by preventing excessive fibrin deposition in acidic environments (61). Notably, fibrin was shown recently to bind to the spike protein of SARS-CoV-2 in the acute-phase of COVID-19, leading to the formation of proinflammatory blood clots that consecutively drive systemic thrombo-inflammation which has the potential to cause neuropathology in COVID-19 (62). Similar anti-inflammatory effects of PTX-3 may be exerted on neutrophils, which are activated for prolonged time in post-acute sequalae of COVID-19 (PASC) (63) and which may be prevented by PTX-3 from rolling on inflamed endothelia/vasculature by binding of PTX-3 to P-selectin and interference with P-selectin glycoprotein ligand (PSGL-1) interaction on neutrophils (64).

Interestingly, a significantly higher percentage of patients with severe COVID-19, compared to those with mild disease, continued to have exhibited high PTX-3 levels even 10 months post-infection. This finding was unexpected, given that during the pandemic elevated PTX-3 levels were widely reported to reflect heightened inflammation and were even proposed as an early biomarker for complications (65) such as secondary pneumonia (15). A return to baseline PTX-3 levels would have been expected by this time point, particularly in our cohort, assessed 10 weeks post-infection. The absence of acute inflammation in these patients was further supported by normal CRP levels (**Figure 1A**), a sensitive clinical indicator of acute infections. Moreover, univariate regression analyses revealed no correlation between PTX-3 and the other assessed acute-phase proteins (**Figures 1F–I**).

What biological mechanism might explain this prolonged elevation of PTX-3 following severe COVID-19?

One potential explanation may involve the persistent presence of non-infectious viral particles (66). While this may be a valid possibility, previous reports claim that only between 2-12% of convalescent patients present with oropharyngeal shedding of SARS-CoV-2 RNA 4 months after SARS-CoV-2 infection and, while around 12.7% [8.5%-18.4%] of patients continued to shed SARS-CoV-2 RNA in the feces at 4 months after diagnosis, this number was reduced to 3.8% [2.0%-7.3%] at 7 months. PTX-3 is typically produced and secreted by innate immune cells in response to pathogen-associated molecular patterns (PAMPs) and damage-associated molecular patterns (DAMPs) (23) but it may also be released by activated endothelial and epithelial cells (28, 29). Thus, it is plausible that in some patients, persistent danger signals trigger sustained PTX-3 expression in the above-mentioned cell types. Furthermore, soluble PTX-3 may interact with the RBD or nucleocapsid (NC) protein of SARS-CoV-2, a function proposed in recent studies (19, 67). However, in our own investigations, no interaction was observed between surface expressed RBD or NC protein (37, 38) and soluble PTX-3.

A second possible explanation for sustained PTX-3 elevation is the influence of host genetics, particularly single nucleotide polymorphisms (SNPs) within the PTX-3 gene. Previous studies have reported that certain SNPs are associated with altered PTX-3 levels, either conferring susceptibility to severe infections due to reduced PTX-3 expression (68) or modulating PTX-3 production in response to LPS stimulation (69). To explore this, we sequenced the *PTX-3* locus, including upstream and downstream regulatory regions. We successfully identified all previously described SNPs along with several novel ones. Notably, SNP rs971145291, located in the 5’ region of *PTX-3*, was heterozygous in all but one patient with a mild disease course. In contrast, the wt A/A genotype was associated with a more severe disease course, as measured both in terms of hospitalization (p<0.0001) (**Figure 4B**) and via a composite score incorporating days of illness, bedridden days, hospitalization and ICU stay (p=0.0051) (**Figure 5**). To better understand the interplay between PTX-3 serum levels, genetic PTX-3 background and disease outcome, we conducted a mediation analysis using a generalized structural equation model (GSEM). This analysis identified PTX-3 serum levels as a complete moderator: The influence of the wt A/A SNP on disease severity was at least partially explained by its effect on PTX-3 serum levels. Sensitivity analyses confirmed the robustness of this relationship, as adjusting for potential confounders did not significantly alter the association (**Figure 5C**).

Another plausible explanation for sustained PTX-3 elevation after severe COVID-19 may involve more extensive tissue damage in the lungs caused by pneumonia and/or mechanical ventilation, all factors leading to more severe epithelial and endothelial damage. This hypothesis is supported by similar factor loadings of PTX-3 with SP-D levels (PC2) but not CRP and SAA levels (PC1) in PCA analyses (**Supplementary Table S2**). This may be explained by the shared biological pathway primarily involving the coordinated production of PTX-3 by immune, endothelial and endothelial cells (9, 25, 29, 70, 71), especially in the lungs in response to infection and tissue injury (72, 73). Instead, the main site of production for CRP and SAA is the liver. PTX-3, in particular, is induced by inflammatory stimuli, which potentially persist due to excessive tissue damage in the aftermath of severe COVID-19. Importantly, elevated levels of PTX-3 and SP-D may reflect not only inflammation but also active tissue repair in the lungs. PTX-3 has been shown to interact with fibrinogen and plasminogen (61), promoting extracellular matrix remodeling and facilitating the clearance of neutrophilic infiltration (73) – both of which are essential for recovery from pulmonary injury. Furthermore, PTX-3 supports the resolution phase by clearing apoptotic cells, a process that may be critical for effective lung repair (61). Moreover, elevated PTX-3 levels may contribute to the resolution of COVID-19-induced microthrombi in the lungs (74, 75), since PTX-3 is known to enhance fibrinolysis (61). Accordingly, PTX-3 may critically contribute to the above-mentioned mechanism of resolving the excessive tissue damage after severe COVID-19 infection and that caused by assisted ventilation, which may be one of the possible reasons, why a considerable number of patients present with high PTX-3 serum levels, as long as 10 weeks post-infection.

The proximal promoter region of the *PTX-3* gene contains multiple enhancer-binding motifs, including sites for Pu-1, AP-1, NF-kB, SP1 and NF-IL-6 (23, 76), which drive expression in response to infection. It is tempting to speculate that the identified SNP (rs971145291) could impair timely transcriptional activation during acute infection. Such a delay may result in an insufficient early immune response, contributing to a more severe disease course. The resulting tissue damage may, in turn, sustain PTX-3 secretion during the convalescent phase, driven by persistent danger signals and ongoing tissue repair processes.

Interestingly, our observations also suggest that PTX-3 may remain high well beyond 10 weeks post-infection in some individuals. Patients with the homozygous wildtype genotype were sampled significantly later (149 ± 59 days versus 103 ± 51 days), yet still showed high PTX-3 levels indicating a possible genetic influence on the duration and/or level of PTX-3 expression (**Supplementary Table S1**). Whether and how the homozygous wt genotype directly contributes to this altered kinetic profile will be the subject of future studies. While most severe COVID-19 patients showed a trend toward normalization of PTX-3 levels at 10 months post-infection, a substantial subset still presented with serum concentrations exceeding 5000 pg/ml (**Figure 3**).

This study has several limitations, the most notable being that patients were assessed at only two discrete timepoints (10w and 10m post-infection) and without baseline evaluation before infection. This limited our ability to analyze intermediate PTX-3 kinetics. In future studies, a more detailed analysis of the PTX-3 kinetics with more frequent sampling time-points, e.g., with monthly serum collections including the baseline time-point, should be undertaken. In addition, a substantial number of severe cases were lost to follow-up between the two visits, limiting longitudinal insights. This is particularly relevant in light of recent studies linking acute-phase PTX-3 levels with the development of long-COVID six months later (60). However, due to the low number of suspected long-COVID-19 cases in our study cohort, this association could not be explored. Moreover, our study protocol only permitted us to ask convalescent patients for COVID-19 related symptoms by a questionnaire at 10w and 10m, so we could not independently confirm, if the few patients, who reported persistent symptoms fulfilled the criteria of long-COVID-19 as defined by WHO (77). Another limitation is that we intentionally included only demographically and sex matched mild cases for sequencing analyses and did not sequence the non-infected controls, which prevented us from assessing the potential influence of certain SNPs on baseline PTX-3 levels. This was due to the fact that our study protocol did not allow for a follow up investigation of the non-infected controls. Moreover, our study was designed as an exploratory study and therefore we did not have the opportunity to perform the genetic analyses for the presence of the SNP rs971145291 in a validation cohort. In future studies, the analysis of non-infected controls for the presence of the SNP and, if possible, follow up data of such individuals on their COVID-19 disease course, would certainly help to better understand the full extent of the contribution of the SNP rs971145291 to disease severity and PTX-3 serum levels. Another possible confounder of our study was the fact that we sampled most of our cases early on during the start of the pandemic, which was caused by the first virus variant (s) (e.g., Wuhan hu-1, alpha). We cannot rule out that later virus variants may have led to different disease outcomes. At these early sampling timepoints, no licensed vaccinations were available and we were therefore unable to investigate whether COVID-19 vaccination alters PTX-3 kinetics or absolute serum levels 10w after a severe disease course. It is undisputed that, SARS-CoV-2 vaccinations, infections with less pathogenic virus variants (e.g., those belonging to the Omega-lineage, etc.) but also the ensuing hybrid immunity have drastically reduced the number of severe COVID-19 cases. Therefore, additional follow up studies in such patients with severe (breakthrough) infections need to be conducted to confirm the selective elevation of PTX-3 10w after infection also in such cases.

In summary, our study identifies PTX-3 as a retrospective marker for severe COVID-19 and further studies are required to investigate its potential usefulness as biomarker for long COVID-19, since PTX-3 is an important regulator of complement activation, tissue remodeling and inflammation. One or more of these processes have been implicated to be dysregulated in long-COVID-19 and a detailed investigation of the role of PTX-3 in long COVID-19 might pave the way for a better understanding of this disease, which is devastating for the afflicted individuals but also for societies due to drastic increases in sick-days and incapacity to work.

## Data Availability

The datasets presented in this study can be found in online repositories. The names of the repository/repositories and accession number(s) can be found in supplements Datasheet 3 (NCBI).
