# Supplementary material for: Severe COVID-19 induces prolonged elevation of the acute-phase protein pentraxin 3
Source: Front Immunol. 2025 Oct 1;16:1672485. doi: 10.3389/fimmu.2025.1672485 (PMC12520919; doi:10.3389/fimmu.2025.1672485)
Supplement: Supplementary file 1 [file DataSheet1.pdf]

Supplementary Material

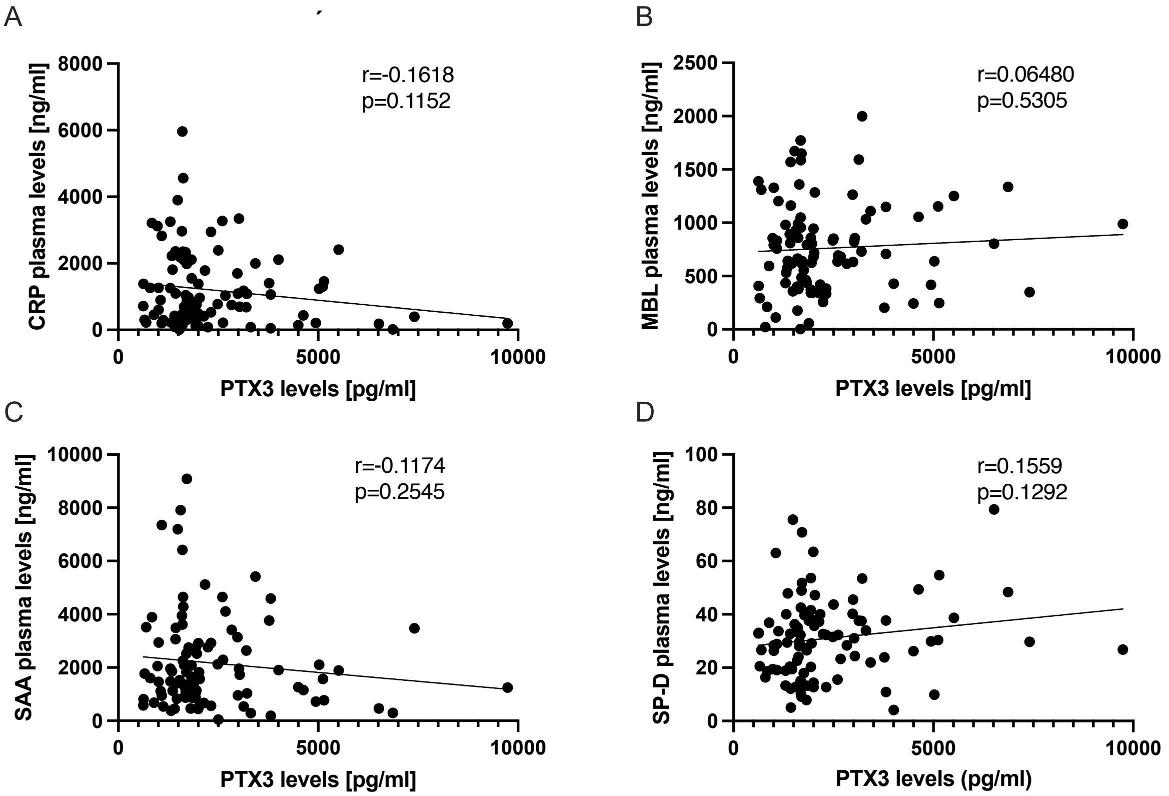

Figure S1: No correlation between PTX-3 plasma levels and the plasma levels of the four other soluble acute phase proteins in non-infected controls. Correlation analyses between the PTX-3 (x-axes) and the plasma levels of (A) CRP (B), MBL (C), SAA and (D) SP-D (y-axes) as determined in the sera of 98 non-infected controls. The Pearson's r and p-values are indicated in the respective plots.

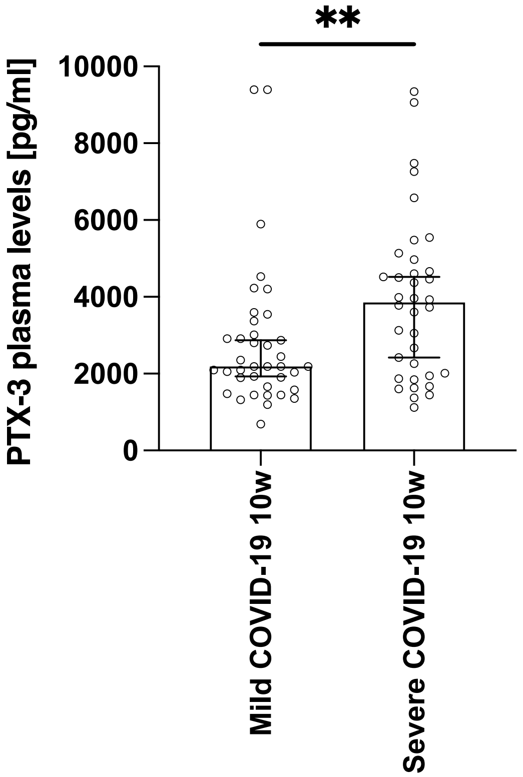

Figure S2: PTX-3 plasma levels of COVID-19 patients who underwent PTX-3 gene sequencing analyses. The PTX-3 plasma levels (median±95%CI; y-axes) of 38 mild and 36 severe COVID-19 cases at 10 weeks. P-value was determined using the Mann Whitney U-test and is indicated as follows: **, $p<0.01$.

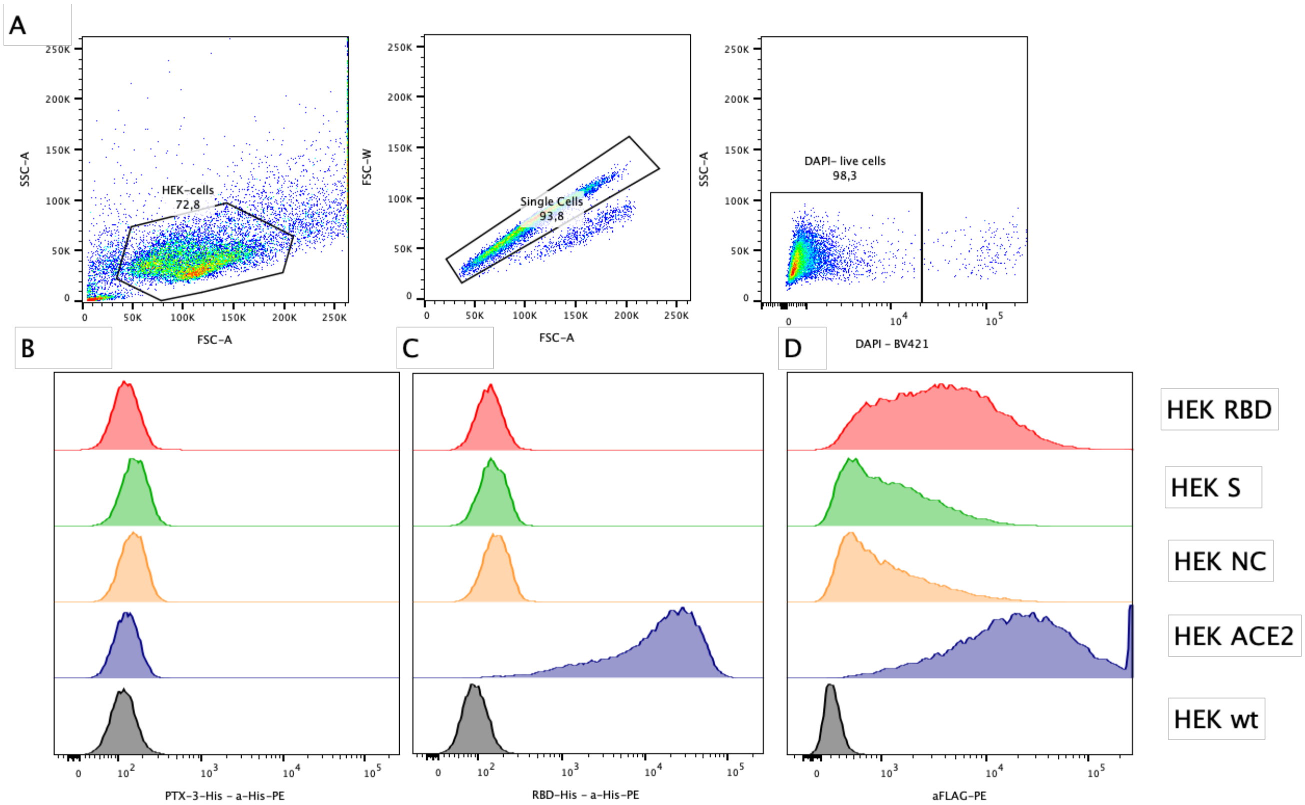

Figure S3: Flow cytometric analyses of PTX-3 binding to transfected HEK cells. (A) Shows the gating strategy for stably transduced HEK cells based on their FSC-SSC properties, selection of single cells (FSC-A vs. FSC-W) and DAPI negative live cells. B-D shows single parameter histogram analyses (x-axes fluorescence intensity, y-axes cell numbers) of indicated HEK transduced cells stained with PTX-3-His and anti-His-PE (B), RBD-His and anti-His-PE (C) as a positive control for anti-His staining and anti-FLAG-PE (D) to confirm expression of the indicated transgenes. Data are representative of three independent experiments.

Table S1. Demographics, clinical presentation and preexisting health conditions of COVID-19 convalescent patients and non-infected subjects.

Characteristics of COVID-19 convalescent patients and non-infected control subjects, p values <0.05 are indicated in red

Characteristics no. (%)	*Non-infected control subjects n=98*	*COVID-19 convalescent patients n=141*	*Mild COVID-19 convalescent patients n=105*	*Severe COVID-19 convalescent patients n=36*	*p-values healthy controls vs COVID-19 convalescent patients*	*p-values mild vs. severe COVID-19 convalescent patients*
Male sex	44 (44.9)	80 (56.7)	59 (56.2)	21 (58.3)	0.0871	0.8481
Female sex	54 (55.1)	61 (43.3)	46 (43.8)	15 (41.6)	0.0871	0.8481
Age Median (Range) (Mean±SD)	51 (14-77) (50 .1±14.2)	54 (16-78) (52.2±14.2)	53 (16-78) (50.8±145)	57.5 (30-78) (56.3±12.5)	0.27561	0.0641
Height in cm (Mean±SD)	172.2±8.9	175.0±9.3	175.5±9.0	173.4±10.4	0.0941	0.4391
Weight in kg (Mean±SD)	73.7±16.4	78.9±16.6	78.3±16.9	80.6±15.6	0.1028	0.2555
Positive SARS-CoV-2 test (rtPCR or serology)	0 (0)	141 (100)	105 (100)	36 (100)	<0.0001	>0.9999
Sneeze	0 (0)	41 (29.1)	27 (25.7)	14 (38.9)	<0.0001	0.1423
Runny nose	0 (0)	47 (33.3)	31 (29.5)	16 (30)	<0.0001	0.1071
Blocked nose	0 (0)	57 (40.4)	43 (41)	14 (38.9)	<0.0001	0.8470
Conjunctivitis	0 (0)	15 (10.6)	14 (13.3)	1 (2.8)	0.005	0.1153
Arthralgia	0 (0)	72 (51.1)	55 (52.4)	17 (47.2)	<0.0001	0.6998
Myalgia	0 (0)	74 (52.5)	57 (54.3)	17 (47.2)	<0.0001	0.5625
Nausea	0 (0)	37 (26.2)	23 (21.9)	14 (38.9)	<0.0001	0.0516
Headache	0 (0)	93 (66.0)	71 (67.6)	22 (61.1)	<0.0001	0.5424
Chills or rigor	0 (0)	73 (51.8)	52 (49.5)	21 (58.3)	<0.0001	0.4405
Fever	0 (0)	112 (79.4)	82 (78.1)	30 (83.3)	<0.0001	0.6350
Max. temperature in °C Median (Range) Mean ± SD	n.a.	38.5 (37.0-42.0) 38.7±0.8	38.5 (37-42) 38.5±0.8	(37.8-41) 39.1±0.8)	n.a.	0.0007
< 37.5°C	n.a.	2 (1.4)	2 (1.9)	0	0.506	>0.9999
37.5 - 38.0°C	n.a.	27 (19.1)	23 (21.9)	4 (11.1)	<0.0001	0.2201
38.1 - 39.0°C	n.a.	51 (36.2)	41 (39-0)	10 (27.8)	<0.0001	0.3149
> 39.0°C	n.a.	33 (23.4)	17 (16.2)	16 (44.4)	<0.0001	0.0011
Fever duration in days (Mean±SD)	n.a.	6.5±5.6	5.6±4.8	9.3±7.0	n.a.	0.0023
Diarrhea	0 (0)	44 (31.2)	28 (26.7)	16 (44.4)	<0.0001	0.0608

Loss of taste/smell	0 (0)	90 (63.8)	68 (64.8)	22 (61.1)	<0.0001	0.6929
Fatigue	0 (0)	119 (84.4)	88 (83.8)	31 (86.1)	<0.0001	>0.9999
Vomiting	0 (0)	16 (11.3)	7 (6.7)	9 (25)	0.0003	0.0054
Other GI problems	0 (0)	10 (7.1)	8 (7.6)	2 (5.6)	0.0059	>0.9999
Sore throat	0 (0)	57 (40.4)	42 (40)	15 (41.7)	<0.0001	>0.9999
Cough	0 (0)	99 (70.2)	73 (69.5)	26 (72.2)	<0.0001	0.8350
Wheezing	0 (0)	43 (30.5)	23 (21.9)	20 (55.6)	<0.0001	0.0003
Shortness of breath	0 (0)	71 (50.4)	44 (41.9)	27 (75)	<0.0001	0.0009
Pneumonia	0 (0)	27 (19.1)	3 (2.9)	24 (66.7)	<0.0001	<0.0001
Days of illness (symptoms)	n.a.	19.4±18.6	15.4±13.1	32.3±26.4	n.a.	<0.0001
Symptom-free days till venipuncture	≥ 70	62.5±34.2	56.5±23.9	81.6±51.7	n.a	0.0158
Days between start of disease and venipuncture	n.a.	84.6 ±39.8	72.8±22.4	119.6±56.6	n.a.	<0.0001
Hospitalized	0 (0)	36 (25.5)	0 (0)	36 (25.5)	<0.0001	<0.0001
Days of hospitalization	n.a.	22.3±20.7	0 (0)	22.3±20.7	n.a.	n.a.
ICU or IMCU admission	0 (0)	16 (11.3)	0 (0)	16 (11.3)	0.0003	<0.0001
Days spent in ICU	n.a.	21.4±11.6	0 (0)	21.4±11.6	n.a.	n.a.
O_2 therapy required	0 (0)	25 (17.7)	1 (1)	24 (17.7)	<0.0001.	<0.0001
Days of O_2 therapy	n.a.	23.7±35.6	28±0	23.4±36.9	n.a.	n.a.
Invasive ventilation	0 (0)	10 (7.1)	0 (0)	10 (7.1)	0.0059	<0.0001
Days of invasive ventilation	n.a.	23.1±5.4	0 (0)	23.1±5.4	n.a.	n.a.
Asymptomatic	98 (100)	5 (3.5)	5 (3.5)	0 (0)	<0.0001	0.3287
Pre-existing health conditions	64 (65.3)	88 (62.4)	65 (61.9)	23 (63.9)	0.6833	>0.9999
Cardiovascular diseases	19 (19.4)	27 (19.1)	20 (19)	7 (19.4)	>0.9999	>0.9999
Chronic lung diseases	11 (11.2)	18 (12.8)	12 (11.4)	6 (16.7)	0.8411	0.4001
Allergy/Asthma	43 (43.9)	45 (31.9)	34 (32.4)	11 (30.6)	0.0761	>0.9999
Diabetes mellitus	4 (4.1)	9 (6.4)	4 (3.8)	5 (13.9)	0.5672	0.0474
Hematopoietic diseases	0 (0)	3 (2.1)	3 (2.9)	0 (0)	0.2712	0.5702
Immunosuppressive conditions	2 (2.0)	8 (5.7)	3 (2.9)	5 (13.9)	0.2048	0.0260
Liver diseases	3 (3.1)	3 (2.1)	3 (2.9)	0 (0)	0.6914	0.5702
Metabolic diseases	17 (17.3)	24 (17.0)	19 (18.1)	5 (13.9)	>0.9999	0.7975
Neurological disorders	8 (8.1)	9 (6.4)	8 (7.6)	1 (2.8)	0.6171	0.4478
Renal diseases	3 (3.1)	5 (3.5)	4 (3.8)	1 (2.8)	>0.9999	>0.9999
Other medical problems	17 (17.3)	32 (22.7)	17 (16.2)	15 (41.1)	0.3335	0.0027
Pregnancy	0 (0)	0 (0)	0 (0)	0 (0)	>0.9999	>0.9999
Smoking	19 (19.4)	9 (6.4)	7 (6.7)	2 (5.6)	0.0036	>0.9999

Regular alcohol consumption	36 (36.7)	55 (39.0)	39 (37.1)	16 (44.4)	0.7869	0.4375
Regular medications	48 (49.0)	72 (51.1)	43 (41)	29 (80.6)	0.7932	<0.0001

6

Table S2: Result of principal component analyses of the relationship of acute phase proteins.

Variable	Loading on Factor 1 (PC1)	Loading on Factor 2 (PC 2)
MBL	0.466821	-0.539976
SP-D	0.234044	0.707217
CRP	0.845570	0.053085
SAA	0.800148	0.129381
PTX3	0.030947	0.703239
**Proportion of total variance explained:**	**0.325776**	**0.261167**

Table S3. Demographics, clinical presentation and preexisting health conditions of mild and severe COVID-19 patients for sequencing analyses

Characteristics of COVID-19 convalescent patients, p values <0.05 are indicated in red

Characteristics no. (%)	*Mild COVID-19 convalescent patients n=38*	*Severe COVID-19 convalescent patients n=36*	*p-values*
Male sex	21 (55.2)	21 (58.3)	0.8148
Female sex	17 (44.7)	15 (41.6)	0.8148
Age Median (Range) (Mean±SD)	56 (30-76) (55.2±11.0)	57.5 (30-78) (56.3±12.5)	0.960
Height in cm (Mean±SD)	175.6±9.5	173.4±10.4	0.4686
Weight in kg (Mean±SD)	79.5±14.3	80.6±15.6	0.6140
Positive SARS-CoV-2 test (rtPCR or serology)	38 (100)	36 (100)	>0.999
Sneeze	8 (21.1)	14 (38.9)	0.1279
Runny nose	10 (26.3)	16 (30)	0.1442
Blocked nose	15 (39.4)	14 (38.9)	>0.999
Conjunctivitis	4 (10.5)	1 (2.8)	0.3585
Arthralgia	23 (60.5)	17 (47.2)	0.3508
Myalgia	25 (65.8)	17 (47.2)	0.1588
Nausea	10 (26.3)	14 (38.9)	0.3222
Headache	28 (73.7)	22 (61.1)	0.3222
Chills or rigor	23 (60.5)	21 (58.3)	>0.999
Fever	35 (92.1)	30 (83.3)	0.3024
Max. temperature in °C Median (Range) Mean ± SD	38.5 (37-42) 38.5±0.7	(37.8-41) 39.1±0.8)	0.0526
< 37.5°C	5 (13.2)	0	0.0546
37.5 - 38.0°C	6 (15.8)	4 (11.1)	0.7366
38.1 - 39.0°C	18 (47.4)	10 (27.8)	0.0979
> 39.0°C	9 (23.7)	16 (44.4)	0.0852
Fever duration in days (Mean±SD)	6.9±5.6	9.3±7.0	0.0410
Diarrhea	9 (23.7)	16 (44.4)	0.0852
Loss of taste/smell	26 (68.4)	22 (61.1)	0.6274
Fatigue	35 (92.1)	31 (86.1)	0.4737
Vomiting	4 (10.5)	9 (25)	0.1319
Other GI problems	4 (10.5)	2 (5.6)	0.6749
Sore throat	17 (44.7)	15 (41.7)	0.8184
Cough	32 (84.2)	26 (72.2)	0.2641
Wheezing	12 (31.6)	20 (55.6)	0.0598
Shortness of breath	19 (50.0)	27 (75)	0.0328
Pneumonia	3 (7.9)	24 (66.7)	<0.0001
Days of illness (symptoms)	15.6±11.0	32.3±26.4	<0.0001
Symptom-free days till venipuncture	57.5±32.7	81.6±51.7	0.0989
Days between start of disease and venipuncture	73.4±29.2	119.6±56.6	0.0002
Hospitalized	0 (0)	36 (25.5)	<0.0001
Days of hospitalization	0 (0)	22.3±20.7	n.a
ICU or IMCU admission	0 (0)	16 (11.3)	<0.0001
Days spent in ICU	0 (0)	21.4±11.6	n.a.
O_2 therapy required	1 (2.6)	24 (17.7)	<0.0001
Days of O_2 therapy		23.4±36.9	n.a.
Invasive ventilation	0 (0)	10 (7.1)	0.0004
Days of invasive ventilation	0 (0)	23.1±5.4	n.a.
Asymptomatic	0 (0)	0 (0)	>0.9999

Pre-existing health conditions	25 (65.8)	23 (63.9)	>0.9999
Cardiovascular diseases	8 (21.1)	7 (19.4)	>0.9999
Chronic lung diseases	3 (7.9)	6 (16.7)	0.3024
Allergy/Asthma	14 (36.8)	11 (30.6)	0.6282
Diabetes mellitus	2 (5.2)	5 (13.9)	0.2553
Hematopoietic diseases	2 (5.2)	0 (0)	0.4935
Immunosuppressive conditions	1 (2.6)	5 (13.9)	0.1027
Liver diseases	1 (2.6)	0 (0)	>0.9999
Metabolic diseases	6 (15.8)	5 (13.9)	>0.9999
Neurological disorders	2 (5.2)	1 (2.8)	>0.9999
Renal diseases	1 (2.6)	1 (2.8)	>0.9999
Other medical problems	9 (23.7)	15 (41.1)	0.1368
Pregnancy	0 (0)	0 (0)	>0.9999
Smoking	1 (2.6)	2 (5.6)	0.6096
Regular alcohol consumption	10 (26.3)	16 (44.4)	0.1442
Regular medications	19 (50)	29 (80.6)	0.0076

Table S4. SNPs detected in the sequenced study cohort

Number of sequenced patients positive for the indicated SNP, p values <0.05 are indicated in red:

Reference SNP ID	Position in the reference genome	COVID-19 convalescent patients (n) n=74	Mild COVID-19 convalescent patients (n) n=38	Severe COVID-19 convalescent patients (n) n=36	p-values (not adjusted) between mild and severe convalescent patients	p-values (corrected for multiple testing) between mild and severe convalescent patients
**rs1191991761**	157431846	1	1	0	>0.9999	>0.9999
**rs899938734**	157431848	4	2	2	>0.9999	>0.9999
**rs1264552796**	157431871	6	2	4	0.4238	>0.9999
**rs4680363**	157432293	53	26	27	0.6105	>0.9999
**rs2316710**	157432391	55	27	28	0.5987	>0.9999
**rs2316709**	157432458	55	27	28	0.5987	>0.9999
**rs1577641602**	157432485	1	1	0	>0.9999	>0.9999
**rs2316708**	157432510	55	27	28	0.5987	>0.9999
**rs2316707**	157432543	2	1	1	>0.9999	>0.9999
**rs1055497867**	157432850	60	29	31	0.3769	>0.9999
**rs80027448**	157432968	1	1	0	>0.9999	>0.9999
**rs549344673**	157433258	2	1	1	>0.9999	>0.9999
**rs971145291**	157434327	58	37	21	<0.0001	<0.0001
**rs968734277**	157434514	60	29	31	0.3769	>0.9999
**rs7615621**	157434811	55	27	28	0.5987	>0.9999
**rs1267892655**	157435080	5	5	0	0.0546	>0.9999
**rs1219400881**	157436137	53	26	27	0.6105	>0.9999
**rs35948036**	157436979	1	1	0	>0.9999	>0.9999
**rs2305619**	157437072	55	27	28	0.5987	>0.9999
**rs1733679197**	157437338	1	0	1	0.4865	>0.9999
**rs3816527**	157437525	60	29	31	0.3769	>0.9999
**rs148943471**	157437630	1	0	1	0.4865	>0.9999
**rs774167944 [**	157437696	2	0	2	0.2332	>0.9999
**rs1372487918**	157437816	1	1	0	>0.9999	>0.9999
**rs781700719**	157438037	3	2	1	>0.9999	>0.9999
**s1200916094**	157438054	3	1	2	0.6069	>0.9999
**rs56025932**	157438069	2	1	1	>0.9999	>0.9999
**rs1840680**	157438240	55	27	28	0.5987	>0.9999
**rs1733831197**	157438261	2	2	0	0.4935	>0.9999
**rs1280296909**	157438623	3	2	1	>0.9999	>0.9999
**rs1044752871**	157441356	61	29	32	0.2236	>0.9999
**rs2109234988**	157441821	0	0	0	>0.9999	>0.9999
**rs2109235022**	157441823	2	1	1	>0.9999	>0.9999
**rs3845978**	157441905	2	1	1	>0.9999	>0.9999
