# Supplementary material for: Severe COVID-19 induces prolonged elevation of the acute-phase protein pentraxin 3
Source: Front Immunol. 2025 Oct 1;16:1672485. doi: 10.3389/fimmu.2025.1672485 (PMC12520919; doi:10.3389/fimmu.2025.1672485)
Supplement: Supplementary file 2 [file DataSheet3.pdf]

Accession	Sample Name	SPUID	Organism	Tax ID	Isolate
SAMN50493007	A064	A064	Homo sapiens	9606	Blood
SAMN50493008	A095	A095	Homo sapiens	9606	Blood
SAMN50493009	A096	A096	Homo sapiens	9606	Blood
SAMN50493010	B002	B002	Homo sapiens	9606	Blood
SAMN50493011	B005	B005	Homo sapiens	9606	Blood
SAMN50493012	B007	B007	Homo sapiens	9606	Blood
SAMN50493013	B009	B009	Homo sapiens	9606	Blood
SAMN50493014	B011	B011	Homo sapiens	9606	Blood
SAMN50493015	B017	B017	Homo sapiens	9606	Blood
SAMN50493016	B018	B018	Homo sapiens	9606	Blood
SAMN50493017	B020	B020	Homo sapiens	9606	Blood
SAMN50493018	B021	B021	Homo sapiens	9606	Blood
SAMN50493019	B022	B022	Homo sapiens	9606	Blood
SAMN50493020	B023	B023	Homo sapiens	9606	Blood
SAMN50493021	B024	B024	Homo sapiens	9606	Blood
SAMN50493022	B026	B026	Homo sapiens	9606	Blood
SAMN50493023	B029	B029	Homo sapiens	9606	Blood
SAMN50493024	B030	B030	Homo sapiens	9606	Blood
SAMN50493025	B037	B037	Homo sapiens	9606	Blood
SAMN50493026	B038	B038	Homo sapiens	9606	Blood
SAMN50493027	B039	B039	Homo sapiens	9606	Blood
SAMN50493028	B040	B040	Homo sapiens	9606	Blood
SAMN50493029	B042	B042	Homo sapiens	9606	Blood
SAMN50493030	B043	B043	Homo sapiens	9606	Blood
SAMN50493031	B050	B050	Homo sapiens	9606	Blood
SAMN50493032	B053	B053	Homo sapiens	9606	Blood
SAMN50493033	B060	B060	Homo sapiens	9606	Blood
SAMN50493034	B063	B063	Homo sapiens	9606	Blood
SAMN50493035	B065	B065	Homo sapiens	9606	Blood
SAMN50493036	B066	B066	Homo sapiens	9606	Blood

SAMN50493037	B074	B074	Homo sapiens	9606	Blood
SAMN50493038	B078	B078	Homo sapiens	9606	Blood
SAMN50493039	B079	B079	Homo sapiens	9606	Blood
SAMN50493040	B081	B081	Homo sapiens	9606	Blood
SAMN50493041	B085	B085	Homo sapiens	9606	Blood
SAMN50493042	B087	B087	Homo sapiens	9606	Blood
SAMN50493043	B089	B089	Homo sapiens	9606	Blood
SAMN50493044	B090	B090	Homo sapiens	9606	Blood
SAMN50493045	B091	B091	Homo sapiens	9606	Blood
SAMN50493046	B093	B093	Homo sapiens	9606	Blood
SAMN50493047	B094	B094	Homo sapiens	9606	Blood
SAMN50493048	B100	B100	Homo sapiens	9606	Blood
SAMN50493049	B121_PTX1-3	B121_PTX1-3	Homo sapiens	9606	Blood
SAMN50493050	B122_PTX1-3	B122_PTX1-3	Homo sapiens	9606	Blood
SAMN50493051	B123_PTX1-3	B123_PTX1-3	Homo sapiens	9606	Blood
SAMN50493052	B134_PTX1-3	B134_PTX1-3	Homo sapiens	9606	Blood
SAMN50493053	B135_PTX1-3	B135_PTX1-3	Homo sapiens	9606	Blood
SAMN50493054	B139_PTX1-3	B139_PTX1-3	Homo sapiens	9606	Blood
SAMN50493055	B144_PTX1-3	B144_PTX1-3	Homo sapiens	9606	Blood
SAMN50493056	B145_PTX1-3	B145_PTX1-3	Homo sapiens	9606	Blood
SAMN50493057	B146_PTX1-3	B146_PTX1-3	Homo sapiens	9606	Blood
SAMN50493058	B147_PTX1-3	B147_PTX1-3	Homo sapiens	9606	Blood
SAMN50493059	B148_PTX1-3	B148_PTX1-3	Homo sapiens	9606	Blood
SAMN50493060	B149_PTX1-3	B149_PTX1-3	Homo sapiens	9606	Blood
SAMN50493061	B152_PTX1-3	B152_PTX1-3	Homo sapiens	9606	Blood
SAMN50493062	B154_PTX1-3	B154_PTX1-3	Homo sapiens	9606	Blood
SAMN50493063	B155_PTX1-3	B155_PTX1-3	Homo sapiens	9606	Blood
SAMN50493064	B156_PTX1-3	B156_PTX1-3	Homo sapiens	9606	Blood
SAMN50493065	B157_PTX1-3	B157_PTX1-3	Homo sapiens	9606	Blood
SAMN50493066	B158_PTX1-3	B158_PTX1-3	Homo sapiens	9606	Blood
SAMN50493067	B162_PTX1-3	B162_PTX1-3	Homo sapiens	9606	Blood

SAMN50493068	B172_PTX1-3	B172_PTX1-3	Homo sapiens	9606	Blood
SAMN50493069	B176_PTX1-3	B176_PTX1-3	Homo sapiens	9606	Blood
SAMN50493070	B185_PTX1-3	B185_PTX1-3	Homo sapiens	9606	Blood
SAMN50493071	B186_PTX1-3	B186_PTX1-3	Homo sapiens	9606	Blood
SAMN50493072	B187_PTX1-3	B187_PTX1-3	Homo sapiens	9606	Blood
SAMN50493073	B188_PTX1-3	B188_PTX1-3	Homo sapiens	9606	Blood
SAMN50493074	B189_PTX1-3	B189_PTX1-3	Homo sapiens	9606	Blood
SAMN50493075	B192_PTX1-3	B192_PTX1-3	Homo sapiens	9606	Blood
SAMN50493076	B193_PTX1-3	B193_PTX1-3	Homo sapiens	9606	Blood
SAMN50493077	B194_PTX1-3	B194_PTX1-3	Homo sapiens	9606	Blood
SAMN50493078	B195_PTX1-3	B195_PTX1-3	Homo sapiens	9606	Blood
SAMN50493079	B196_PTX1-3	B196_PTX1-3	Homo sapiens	9606	Blood
SAMN50493080	B197__PTX1-3	B197__PTX1-3	Homo sapiens	9606	Blood

Object IDs and corresponding URLs:

50493007: https://www.ncbi.nlm.nih.gov/sra/50493007

50493008: https://www.ncbi.nlm.nih.gov/sra/50493008

50493009: https://www.ncbi.nlm.nih.gov/sra/50493009

50493010: https://www.ncbi.nlm.nih.gov/sra/50493010

50493011: https://www.ncbi.nlm.nih.gov/sra/50493011

50493012: https://www.ncbi.nlm.nih.gov/sra/50493012

50493013: https://www.ncbi.nlm.nih.gov/sra/50493013

50493014: https://www.ncbi.nlm.nih.gov/sra/50493014

50493015: https://www.ncbi.nlm.nih.gov/sra/50493015

50493016: https://www.ncbi.nlm.nih.gov/sra/50493016

50493017: https://www.ncbi.nlm.nih.gov/sra/50493017

50493018: https://www.ncbi.nlm.nih.gov/sra/50493018

50493019: https://www.ncbi.nlm.nih.gov/sra/50493019

50493020: https://www.ncbi.nlm.nih.gov/sra/50493020

50493021: https://www.ncbi.nlm.nih.gov/sra/50493021

50493022: https://www.ncbi.nlm.nih.gov/sra/50493022

50493023: https://www.ncbi.nlm.nih.gov/sra/50493023

50493024: https://www.ncbi.nlm.nih.gov/sra/50493024

50493025: https://www.ncbi.nlm.nih.gov/sra/50493025

50493026: https://www.ncbi.nlm.nih.gov/sra/50493026

50493027: https://www.ncbi.nlm.nih.gov/sra/50493027

50493028: https://www.ncbi.nlm.nih.gov/sra/50493028

50493029: https://www.ncbi.nlm.nih.gov/sra/50493029

50493030: https://www.ncbi.nlm.nih.gov/sra/50493030

50493031: https://www.ncbi.nlm.nih.gov/sra/50493031

50493032: https://www.ncbi.nlm.nih.gov/sra/50493032

50493033: https://www.ncbi.nlm.nih.gov/sra/50493033

50493034: https://www.ncbi.nlm.nih.gov/sra/50493034

50493035: https://www.ncbi.nlm.nih.gov/sra/50493035

50493036: https://www.ncbi.nlm.nih.gov/sra/50493036

50493037: https://www.ncbi.nlm.nih.gov/sra/50493037

50493038: https://www.ncbi.nlm.nih.gov/sra/50493038

50493039: https://www.ncbi.nlm.nih.gov/sra/50493039

50493040: https://www.ncbi.nlm.nih.gov/sra/50493040

50493041: https://www.ncbi.nlm.nih.gov/sra/50493041

50493042: https://www.ncbi.nlm.nih.gov/sra/50493042

50493043: https://www.ncbi.nlm.nih.gov/sra/50493043

50493044: https://www.ncbi.nlm.nih.gov/sra/50493044

50493045: https://www.ncbi.nlm.nih.gov/sra/50493045

50493046: https://www.ncbi.nlm.nih.gov/sra/50493046

50493047: https://www.ncbi.nlm.nih.gov/sra/50493047

50493048: https://www.ncbi.nlm.nih.gov/sra/50493048

50493049: https://www.ncbi.nlm.nih.gov/sra/50493049

50493050: https://www.ncbi.nlm.nih.gov/sra/50493050

50493051: https://www.ncbi.nlm.nih.gov/sra/50493051

50493052: https://www.ncbi.nlm.nih.gov/sra/50493052

50493053: https://www.ncbi.nlm.nih.gov/sra/50493053

50493054: https://www.ncbi.nlm.nih.gov/sra/50493054

50493055: https://www.ncbi.nlm.nih.gov/sra/50493055

50493056: https://www.ncbi.nlm.nih.gov/sra/50493056

50493057: https://www.ncbi.nlm.nih.gov/sra/50493057

50493058: https://www.ncbi.nlm.nih.gov/sra/50493058

50493059: https://www.ncbi.nlm.nih.gov/sra/50493059

50493060: https://www.ncbi.nlm.nih.gov/sra/50493060

50493061: https://www.ncbi.nlm.nih.gov/sra/50493061

50493062: https://www.ncbi.nlm.nih.gov/sra/50493062

50493063: https://www.ncbi.nlm.nih.gov/sra/50493063

50493064: https://www.ncbi.nlm.nih.gov/sra/50493064

50493065: https://www.ncbi.nlm.nih.gov/sra/50493065

50493066: https://www.ncbi.nlm.nih.gov/sra/50493066

50493067: https://www.ncbi.nlm.nih.gov/sra/50493067

50493068: https://www.ncbi.nlm.nih.gov/sra/50493068

50493069: https://www.ncbi.nlm.nih.gov/sra/50493069

50493070: https://www.ncbi.nlm.nih.gov/sra/50493070

50493071: https://www.ncbi.nlm.nih.gov/sra/50493071

50493072: https://www.ncbi.nlm.nih.gov/sra/50493072

50493073: https://www.ncbi.nlm.nih.gov/sra/50493073

50493074: https://www.ncbi.nlm.nih.gov/sra/50493074

50493075: https://www.ncbi.nlm.nih.gov/sra/50493075

50493076: https://www.ncbi.nlm.nih.gov/sra/50493076

50493077: https://www.ncbi.nlm.nih.gov/sra/50493077

50493078: https://www.ncbi.nlm.nih.gov/sra/50493078

50493079: https://www.ncbi.nlm.nih.gov/sra/50493079

50493080: https://www.ncbi.nlm.nih.gov/sra/50493080
